# Design of a 3D-printed, open-source wrist-driven orthosis for individuals with spinal cord injury

**DOI:** 10.1371/journal.pone.0193106

**Published:** 2018-02-22

**Authors:** Alexandra A. Portnova, Gaurav Mukherjee, Keshia M. Peters, Ann Yamane, Katherine M. Steele

**Affiliations:** 1 Department of Mechanical Engineering, University of Washington, Seattle, WA, United States of America; 2 Division of Prosthetics & Orthotics, Department of Rehabilitation Medicine, University of Washington, Seattle, WA, United States of America; Northwestern University, UNITED STATES

## Abstract

Assistive technology, such as wrist-driven orthoses (WDOs), can be used by individuals with spinal cord injury to improve hand function. A lack of innovation and challenges in obtaining WDOs have limited their use. These orthoses can be heavy and uncomfortable for users and also time-consuming for orthotists to fabricate. The goal of this research was to design a WDO with user (N = 3) and orthotist (N = 6) feedback to improve the accessibility, customizability, and function of WDOs by harnessing advancements in 3D-printing. The 3D-printed WDO reduced hands-on assembly time to approximately 1.5 hours and the material costs to $15 compared to current fabrication methods. Varying improvements in users' hand function were observed during functional tests, such as the Jebsen Taylor Hand Function Test. For example, one participant's ability on the small object task improved by 29 seconds with the WDO, while another participant took 25 seconds longer to complete this task with the WDO. Two users had a significant increase in grasp strength with the WDO (13–122% increase), while the other participant was able to perform a pinching grasp for the first time. The WDO designs are available open-source to increase accessibility and encourage future innovation.

## Introduction

Approximately 276,000 individuals in the US have had a spinal cord injury (SCI), 47% of which result in tetraplegia, or some form of paralysis in all four limbs [1]. While both upper- and lower-extremity function is affected in tetraplegia, approximately 75% of these individuals state that they would prefer to regain hand function to any other lost abilities, including bowel, bladder, sexual functions, and the ability to walk [2]. With limited upper-extremity function, these individuals find it challenging to perform activities of daily living (ADLs) such as self-feeding, bathing, dressing, and toileting. As a result, they often require outside assistance in the form of caretakers and assistive devices. To enable independence of individuals with tetraplegia, clinicians and researchers provide tools to help regain or compensate for lost hand function.

**Fig 1:**
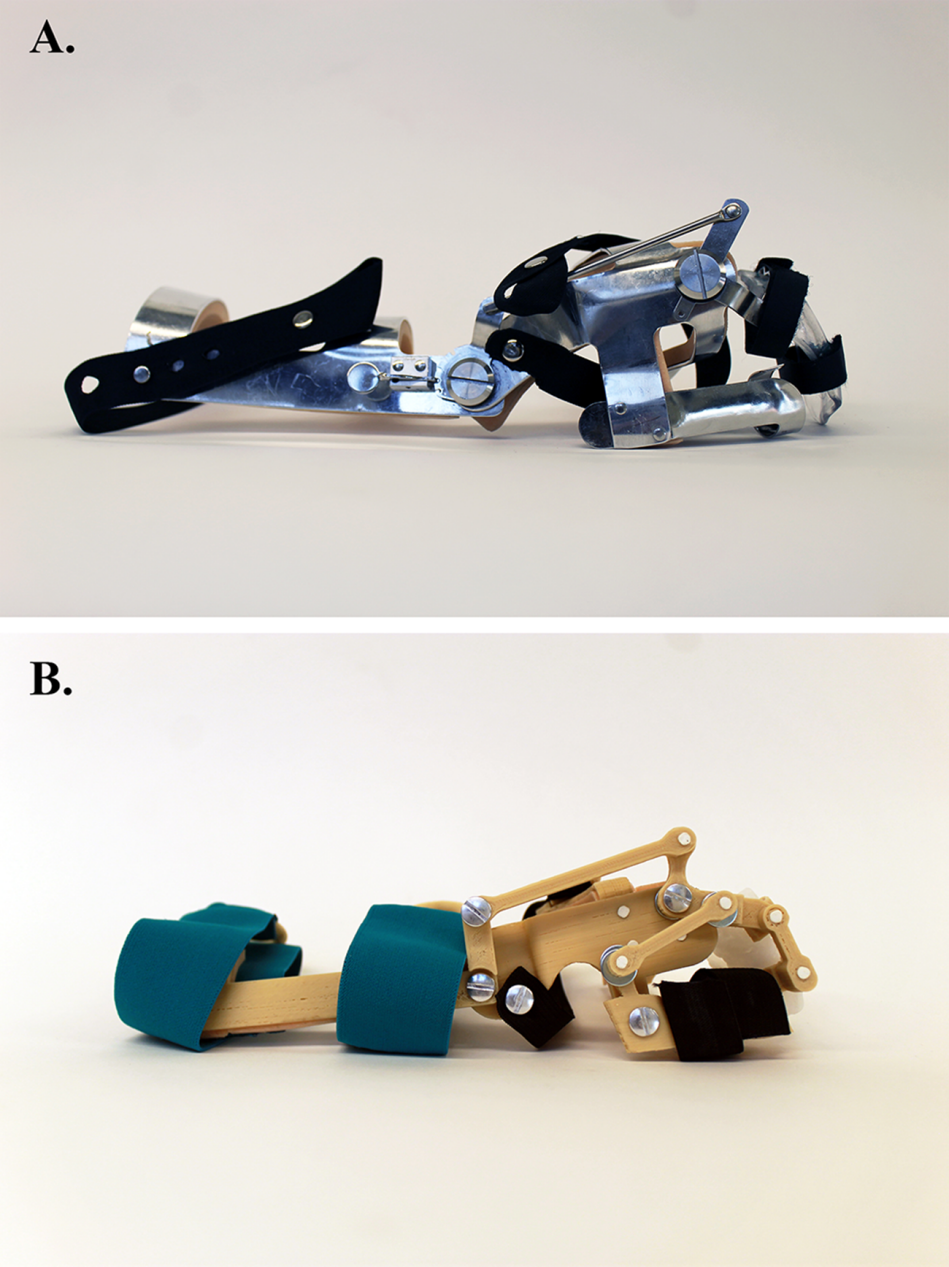
Metal and 3D-printed WDOs. (A) Traditional WDO fabricated from the Jaeco kit. (B) 3D-printed WDO from this research.

One such device is a wrist-driven wrist-hand orthosis (WDO, Fig 1A), designed for individuals with SCI at the 6^th^-7^th^ cervical levels (C6-C7), the most common levels of cervical SCI [3]. With this level of injury, individuals can voluntarily move their wrists but lack finger movement. To use a WDO, an individual must be able to extend their wrist against gravity (manual muscle test, wrist extensor strength ≥ 3). With a stabilized thumb and partially flexed index and middle fingers, WDOs assist in closing the hand with active wrist extension and opening with gravity-assisted wrist flexion using a four-bar linkage mechanism. The WDO imitates a three-jaw chuck grasp, which accounts for 80% of all prehension grasps, including activities such as self-feeding, writing, and hygiene [4]. Unlike static splints that are used to immobilize an injured or displaced body part, WDOs and other upper-extremity orthoses are dynamic and enhance function through a movable mechanism.

While WDOs are often provided to improve hand function in SCI, the number of prior studies that have evaluated performance of ADLs with WDOs and other orthotic devices is limited. For WDOs, one prior study suggested that individuals who use the device for functional tasks throughout the day regain wrist strength and improve prehension [3]. Another study demonstrated significant improvements in pinch force with WDOs that was associated with users’ wrist extensor strength [5]. Research with static wrist splints have shown mixed results. One study demonstrated an increase in pinch force [6] when the wrist was held in extension, but others have demonstrated a significant reduction in both grip strength and wrist range of motion (ROM) compared to function without static devices [7,8]. Research on hand splints have also suggested that splints may increase passive range of motion [9–11] and decrease pain [19,10,12], although these devices are not designed to assist with gripping function like a WDO. There is a clear need for greater functional testing and user feedback in the design and evaluation of orthoses and splints for individuals with SCI.

Despite their potential benefits to individuals with SCI, WDOs are often not provided to potential users due to resource constraints or are frequently abandoned by patients. While abandonment of WDOs has not been studied among individuals with SCI, high rates of abandonment for other hand orthoses for individuals with arthritis or neurologic injuries cite issues with ease of use, function, or dissatisfaction with device appearance [13–16]. One possible solution to these problems could be customization of orthotic devices, ensuring that users are provided with devices that properly address their needs and allow for necessary functionality in daily tasks. Despite its importance, customization poses a variety of challenges to both users and orthotists. While orthotic users often find devices lacking comfort, aesthetics, or necessary function, orthotists struggle with laborious and time-consuming fabrication methods that require exact precision of component sizes [17–19].

In current clinical practice in the United States, WDOs are typically fabricated by certified orthotists using a commercially-available kit (*e*.*g*., Jaeco), which consists of aluminum sheets and additional parts for the four-bar linkage. Orthotists contour the sheets into the desired shape to fit each individual’s arm and assemble the parts using rivets. Thermoplastic material can be used to create a customized finger shell for the second and third fingers. The price for current kits is roughly $140, which does not include the costs associated with the clinicians’ time to assemble, fit, and train the individual to use the device. Reimbursement for clinician time for fabrication and training is highly variable between medical systems. Through surveying orthotists, it was determined that for a hybrid design that includes both metal and plastic fitting, an average orthotist may take around 3 hours for patient’s evaluation, measurements, casting, and fittings, and approximately 8 hours for device fabrication.

In addition to challenges with cost, the dynamic function of WDOs also poses a challenge for patient-specific customization. Unlike splints, WDOs include components, such as ratchet mechanisms, that cannot be easily molded and modified, yet need to be carefully fit to ensure smooth movement and operation of the device. The kits for WDO fabrication come in three different sizes: small, medium, and large, with no options available for pediatric users. Alternatively, clinicians can purchase an off-the-shelf, one-size-fits-all WDO, which typically ranges in price from $350-$750, not including clinician’s time. Nonetheless, the one-size-fits-all approach cannot accommodate for the entire patient population with diverse hand shapes and sizes.

Improvements in the design and fabrication of WDOs have also been limited. Some of the earliest design modifications allowed greater flexibility of fit, such as accommodating individuals with excessive radial deviation by incorporating a ball-joint into the mechanism design without hindering movement [20]. Advances have also been made in the WDO’s driving mechanisms. For example, a functional ratchet system can be added to the WDO to allow the user to passively maintain a closed grasp at different wrist angles [21]. Manufacturers have designed electrically-powered mechanisms (*e*.*g*., PowerGrip, ~$3,600) for individuals with reduced wrist extensor strength, and several recent studies have prototyped other actuated designs, using myoelectric signals or other control methods [22–24]. However, the performance of these designs relative to function without a device or traditional body-powered devices remains unknown.

Recent advances in additive manufacturing (*i*.*e*., 3D-printing) and computer-aided design (CAD) may provide new tools to improve fabrication and accessibility of assistive devices such as WDOs. With their constantly-increasing popularity and accessibility, 3D printers can now be found in clinics, universities, makerspaces, libraries, and even public schools [25–27]. 3D-printing relies heavily on the use of CAD modeling, which, in addition to providing visualization of designs, can be used to modify existing models, test the validity and functionality of created designs, and customize products upon users’ requests. There are many types of 3D-printing, but one of the most common and affordable types, fused filament fabrication (FFF), has been greatly utilized in fabrication and development of assistive devices [28–41]. FFF uses plastic filament that is melted onto a build plate into a desired 2D-shape where it solidifies. After each layer is completed, the build plate moves down to allow for another layer to be printed on top of the previous layer until the desired shape of a 3D-object is formed.

One of the most common application of this method in the field of assistive devices has been to improve prosthetic fabrication and fit. For example, an open-source community, *Enabling the Future*, has developed over 13 hand prosthetic devices to date that are wrist-, elbow-, and myoelectrically-driven [40,42–44]. In addition, 3D-printing has been widely applied to the fabrication of foot [28,30–33] and spine [29] orthoses as well as hand splints [34–36,41]. Nonetheless, the prior work on using FFF to improve mechanically-driven upper-extremity orthoses with more complicated designs, such as the WDO, is limited. Research labs have worked on designing mechanical orthoses similar to the WDO [45] and have integrated an electromyographic controller for the device [23]; however, none have focused on improving the fabrication technique of the existing designs and performed user testing.

The aim of this research was to improve WDO design and fabrication for individuals with SCI by (1) developing and quantifying improvements in WDO fabrication using CAD and 3D-printing technology and (2) iteratively testing the design of a 3D-printed WDO on hand function with SCI users. A human-centered design approach was used as the framework for this research [46–48], in which the research team worked closely with students from a Prosthetics & Orthotics (P&O) program and members of the local SCI community to create a 3D-printed WDO design that addresses important functional, design, and fabrication challenges (Fig 1B).

## Materials and methods

### Device design

The research team for this study consisted of engineers, certified orthotists, and a kinesiologist. In collaboration with local orthotists, a list of design requirements was created for the WDO, including wearability, aesthetics, fit/adjustability, functionality, weight, customizability, durability, and modularity (Table 1). Using these design requirements, a model of the WDO was created in CAD software (SolidWorks, Waltham, MA). Each component of the WDO was designed such that the dimensions could be easily manipulated to fit multiple users. Only a subset of dimensions for each part varied with size, while all others were fixed. CAD software was used throughout the process to iterate on the design based upon the P&O students’ and users’ feedback.

**Table 1:** Design requirements for the WDO.

*Design Requirements*	*Description*
***Wearability***	The device must be comfortable to wear for extended use and easy to don and doff.
***Fit & Adjustability***	Each device component must allow scaling for different hand shapes and sizes and come in right and left versions. Straps should allow flexibility in fit and comfort.
***Functionality***	The device must be intuitive to use and enable a three-jaw chuck grasp.
***Weight***	The device must not exceed 150 grams, the weight of traditional metal WDOs.
***Aesthetics***	The design should allow customizable colors of device components.
***Modularity***	Each component must be easily replaceable or repairable.

An affordable and widely-available FFF 3D-printer was used to fabricate the WDO parts (FlashForge Creator Pro, FlashForge, Rowland Heights, CA). To prepare the CAD models for printing, a free slicing software (MakerBot Desktop, New York, NY) was used to slice the model into thin layers and position it properly onto the build plate (see p. 11 of S1 Manual). Parts were printed using 1.75mm polylactic acid (PLA) plastic filament. PLA was chosen because of its affordability, accessibility, and ease of use. The print settings were 30% hexagonal infill, 4 shells, supports, and rafts. Individual parts were oriented on the build plate in the direction that would result in minimum support material for smoother surface finish. A step-by-step manual (S1 Manual) was created to guide printing and assembly of the WDO, including the process of obtaining necessary hand measurements with a tape measure (see p.4-7 of S1 Manual) and inputting correct dimensions into the CAD software to ensure a customized fit for various hand shapes and sizes (see p.6,8–10 of S1 Manual). After feedback from P&O students and users, the CAD models and instruction manual were updated to integrate recommended improvements. All testing with orthotists and users was approved by the University of Washington’s Institutional Review Board (IRB). Informed written consent was obtained from each study participant.

### Fabrication testing

**Fig 2:**
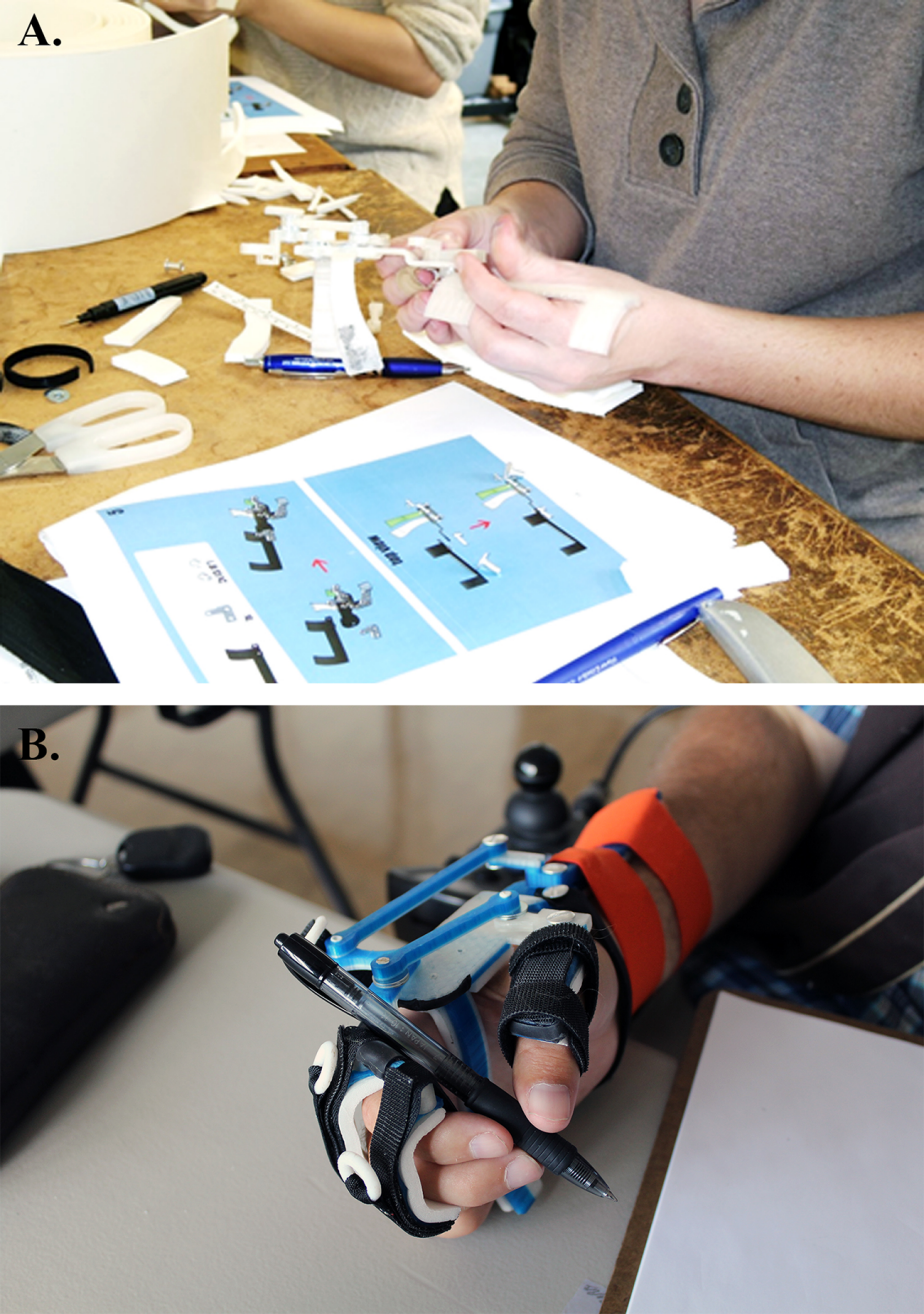
The experimental setup of the study with 3D-printed WDOs. (A) P&O student fabricating a 3D-printed WDO using the device manual. (B) User performs the writing task of the JTHF test with a 3D-printed WDO.

To test the ease of fabrication of the 3D-printed WDO as well as its comfort, aesthetics, and function, a convenience sample of six P&O students from the University of Washington were recruited (Fig 2A). All P&O students participating in this study had prior experience fabricating traditional metal WDOs as part of their education. Students in the P&O program at this institution have also received a basic introduction to CAD and 3D-printing as part of their curriculum. The students were separated into two groups and each group participated in one visit to fabricate 3D-printed WDOs.

At each visit, the P&O students were provided with the instruction manual containing visual and written instructions that outlined the steps of orthosis fabrication and assembly. These instructions helped provide the background for 3D-printing and WDO fabrication. Each student was presented with 10-minutes of instruction on how to manipulate a model of the orthosis on the computer and set parameters for 3D-printing. Afterwards, they were asked to assemble the WDO using the manual, pre-printed components, as well as needed materials and tools (S1 Manual). The process of assembly was timed, and outside help and clarification from the research team were provided when needed. Additional help provided to the P&O students during this testing was minimal and included clarifications of the assembly steps in the manual. Members of the research team observed the P&O students and noted which parts of the assembly process they struggled/succeeded with.

After assembly, the P&O students were asked to fill out a survey about their experience with assembling a 3D-printed WDO compared to traditional metal WDOs. In the survey, they also rated the 3D-printed WDO on a scale from 1 (slow/poor) to 10 (fast/great) in terms of fabrication speed, perceived function, aesthetics, and comfort for an individual with a cervical SCI (S1 Survey). Lastly, they were asked to provide feedback on possible improvements in the fabrication process and WDO design. As part of the human-centered design process, their questions and suggestions were used to modify and improve the WDO and instructions. Design and fabrication improvements of the WDO were made after each group’s visit.

### User testing

To evaluate the performance and usability of the 3D-printed WDO, the device was tested with individuals with SCI (Fig 2B). Three individuals with prior cervical SCI (Table 2) who had little to no mobility in their fingers, but were able to extend their wrists against gravity, were recruited to participate in this study. Individuals with skin abrasions, sensitivity on the affected limb, or lack of cognitive ability to follow instructions were excluded.

**Table 2:** Participant characteristics.

*User*	*Age* (yr)	*Sex*	*Level of Injury*	*Type of Injury*	*Years Since Injury*	*Dominant Hand*	*Device Hand*	*Prior Experience with WDO*?
***P1***	40	M	C5, C6	complete	16	right	right	yes
***P2***	54	F	C5, C6	incomplete	28	right	left	yes
***P3***	65	F	C4, C6	incomplete	18.5	right	left	no

During the first visit, measurements were taken of the user’s hand to determine the appropriate size of the 3D-printed WDO (see p.7 of S1 Manual). The list of measurements was optimized to minimize the time required while ensuring a close match between the WDO’s joints and the hand joints (*i*.*e*., wrist, metacarpophalangeal joints) to improve function.

During the second visit, users were fit with their custom 3D-printed WDO. The user’s ability to don and doff the WDO (*i*.*e*., putting the device on and taking it off) was observed by measuring both the time required and qualitative comments from the user. Each user’s pain and motivation levels were recorded using a self-reported 1–10 scale before using the WDO. Custom modifications, such as additional padding, change of strap lengths, and tightness of screws on the device, were performed to customize the fit to each user and ensure high comfort levels during testing. Each user was given approximately 10–15 minutes of acclimatization time with the device during which they attempted to pick up water bottles, pens, and other objects with the 3D-printed WDO.

Hand function with and without the WDO was evaluated using the Box-and-Blocks Test, Jebsen-Taylor-Hand-Function (JTHF) Test, and pinch dynamometry (S1 Video). These tests were chosen based upon tests currently used by our clinical partners and in prior research studies. During the Box-and-Blocks Test, users were asked to transfer blocks as quickly as possible, one at a time from one side of the standardized box to the other over a partition for one minute. Each user was given a 15-second practice time. During the JTHF Test, the users were asked to perform a series of tasks simulating ADLs as fast as possible (*i*.*e*., writing, card turning, picking up small objects, stacking checkers, simulated feeding, and moving large light and heavy objects). Using a pinch dynamometer, the maximum pinch force generated between the fingers during a three-jaw chuck grasp was recorded. Each user was asked to pinch the device as hard as possible with verbal encouragement over approximately 5 seconds for three trials. The order of the tests varied across the participants, but was not formally randomized. The variation was done to prevent fatigue from each test, motivate participants during each session, and provide significant breaks between tasks.

Upon completing the hand function tests, the users were asked to rate the 3D-printed WDO in terms of its function, aesthetics, comfort, ease of donning, and appropriateness for their needs on a scale from 1 (poor) to 10 (great). In addition, they were asked about prior experience with traditional hand orthoses and asked for suggestions on how to improve the current design. These comments were audio-recorded and transcribed to identify common themes and suggestions. The user-suggested modifications were incorporated into the design for further testing. Two users (P2 and P3) returned for follow-up visits to perform the hand function tests with modified designs.

## Results

### Device design

**Fig 3:**
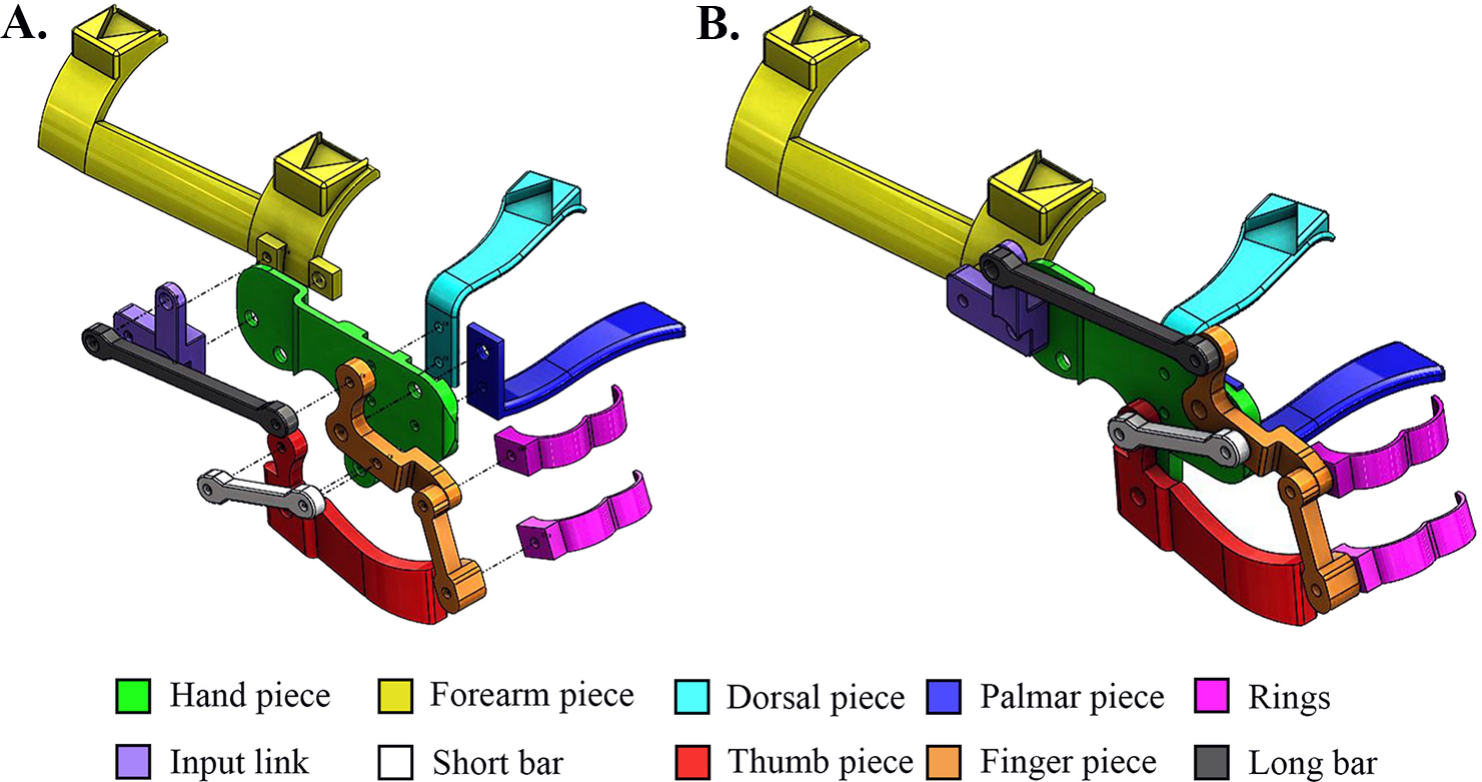
**Exploded (A) and assembled (B) view of the CAD model of the 11 pieces of the 3D-printed WDO**.

A 3D-printed WDO was designed based upon user and orthotist feedback. The final design consists of 11 parts: hand, forearm, palmar and dorsal pieces, long and short bars, input link, thumb and finger pieces and two finger rings (Fig 3). The parts are assembled at the joints using five aluminum post-screws and eight Acrylonitrile Butadiene Styrene (ABS) pins, a plastic similar to PLA, in the non-load-bearing joints. The original design contained aluminum post-screws for each joint, increasing the weight and profile of the device. However, ABS pins were found to be a light-weight, low-profile alternative. Post-screws are tightened and secured with a thread-locking adhesive to prevent loosening. To create ABS pins, a length of ABS filament is inserted into each joint. Heat is applied at one end with a soldering iron and is pressed against a flat surface to create a flattened constraint. The opposite end of the ABS filament is cut to an appropriate length, with about 0.1” of extra filament, and is similarly flattened to the back of the 3D-printed part (see p. 20–21 of S1 Manual).

The SolidWorks files for each WDO component can either be custom-sized for an individual or printed in one of five available sizes: XS, S, M, L, and XL. These sizes were designed for users with limited background in CAD modeling using hand anthropometry data compiled by the Georgia Tech Research Institute [49]. These sizes, however, are not able to provide absolute fit. For more experienced users, the manual provides instructions that explain which CAD dimensions of each component should be modified in accordance with the hand measurements of the user. These instructions are included in the device manual (see p. 7–10 of S1 Manual).

**Fig 4:**
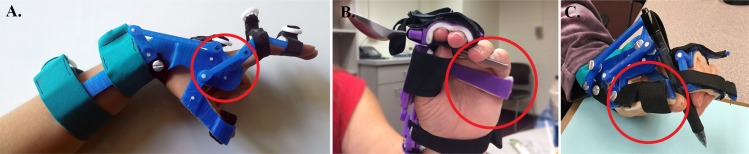
Selected design improvements in the 3D-printed WDO. (A) Additional linkage between the thumb and the finger piece. (B) Curved thumb piece. (C) Elongated palmar piece.

While the main four-bar linkage components from the traditional WDO were preserved in the 3D-printed model, design modifications were also made to improve the comfort and functionality of the device. Notably, a linkage between the thumb and the finger piece was added to the design (Fig 4A). This linkage allowed for a greater hand aperture during full wrist flexion with minimal increase in the design complexity. While the hand aperture was only 52° for full wrist flexion in the original design, it increased to 70° with the additional linkage. In addition, this design modification allowed the fingers and the thumb to open together as in a natural grasp.

Another design modification from user feedback was replacing the Velcro straps used to hold the device in place with elastic straps and magnets to avoid Velcro hooks attaching onto articles of clothing (see p. 29–30 of S1 Manual). Magnet attachments were easier for the users to operate and securely attach. Each strap contains a ring at the end to allow users with flaccid fingers to grab onto them with ease and loop the strap around their hand. Self-adhesive padding was added at the area of contact between the WDO and the user’s skin (see p. 28 of S1 Manual). Moldable plastic (Instamorph) was used as a support material for the second and third digits, ensuring their proper placement for the three-jaw chuck grasp (see p. 32–34 of S1 Manual).

The total device cost was $15 and $20 for XS and XL 3D-printed WDOs, respectively. This includes the cost of all the material used in the fabrication (*e*.*g*., PLA filament, magnets, straps, Velcro). The cost does not include the potential costs associated with using a public 3D-printer and purchasing assembly tools such as heat gun, screwdriver, glue gun, wire cutters, sandpaper, and scissors. Approximate costs of these tools are reported in Table 3. The average assembly time was 8 hours (6.5 hours of hands-off 3D-printing, 1.5 hours of hands-on assembly) and 9.2 hours (7.7 hours of 3D-printing, 1.5 hours of assembly) for XS and XL 3D-printed WDOs, respectively.

**Table 3:** Estimated costs of the equipment and tools used for WDO fabrication and assembly.

*Equipment*	*Approximate Cost*
***3D printer (FlashForge Creator Pro)***	$900.00
***Heat gun***	$60.00
***Screwdriver***	$2.00
***Glue gun***	$25.00
***Wire cutters***	$7.00
***Sandpaper***	$5.00
***Scissors***	$10.00

### Testing with P&O students

**Fig 5:**
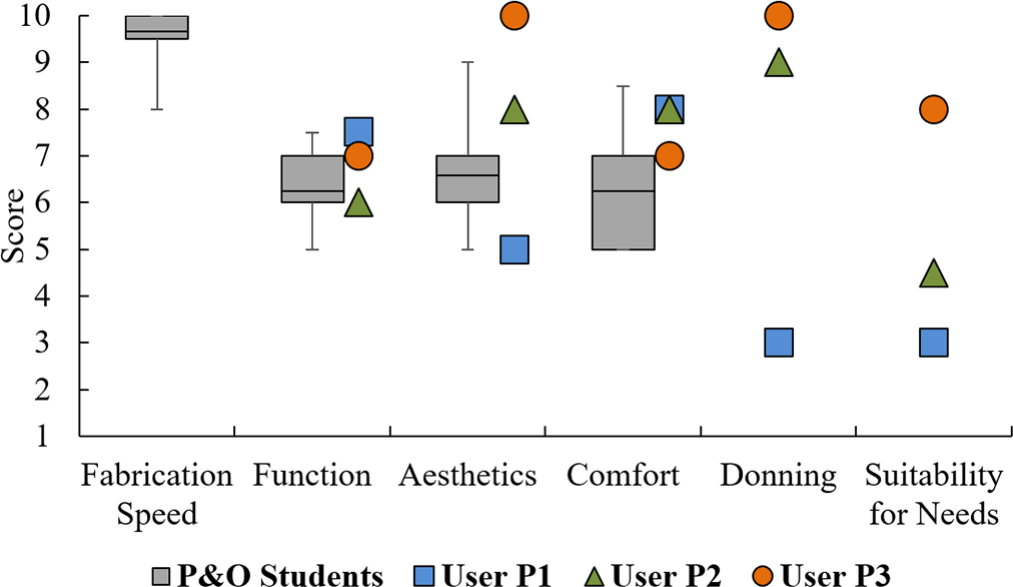
Device scores from testing with P&O students and users. Scores from P&O students (gray boxes) rating the 3D-printed WDO on fabrication speed, function, aesthetics, and comfort (1 = poor/slow, 10 = great/fast). Scores from the three individuals with SCI (colored points) rating the function, aesthetics, comfort, ease of donning, and suitability of the 3D-printed device to their daily needs. Only results from the last visit are shown for each of the users, to account for the iterative modifications to the WDO design.

Testing with the P&O students provided quantitative and qualitative feedback on the fabrication, fit, and function of the WDO. The device received average scores of 6.3, 6.6, 6.3, and 9.7 on function, aesthetics, comfort, and fabrication speed, respectively (Fig 5). The P&O students’ feedback also led to further design improvements that were incorporated for user testing. Noteworthy improvements include a curved thumb piece for a stable three-jaw chuck grasp (Fig 4B), elongated palmar piece for hand stability (Fig 4C), and replacement of aluminum post-screws with ABS pins to reduce the profile of the device (see p. 20–22 of S1 Manual). The P&O students also commented on the “appeal of a 3D-printed WDO in pediatrics” and its potential to “increase availability and accessibility of the device.” One student noted that “the overall experience of 3D-printing an orthosis is great, a lot faster than the metal version.” Despite the positive feedback, many participating students voiced their concern about the durability of the 3D-printed WDO. They have also voiced the potential issues that might arise in the future with billing for devices fabricated in such a way, since these techniques have not yet been widely introduced to the P&O field. A more comprehensive list of P&O students’ comments is included in S1 Table.

### Testing with users

Testing with users provided further important quantitative and qualitative feedback on the 3D-printed WDO. The users became designers, as they offered key insights and suggestions for design improvements based upon their experiences and preferences. The 3D-printed device received average scores of 6.8, 7.7, and 7.7 for function, aesthetics, and comfort from the users (Fig 5), which are higher than those scores from the P&O students. Scores for subjective categories such as “suitability for need” and “aesthetics” had a greater spread among users than those in categories such as “function” and “comfort.” Modifications were made during the visit to accommodate user requests (*e*.*g*., shortening of the thumb piece, addition of padding). Other modifications were performed in the lab and applied to the overall design of the device (*e*.*g*., replacement of Velcro straps with magnet strap attachments).

**Fig 6:**
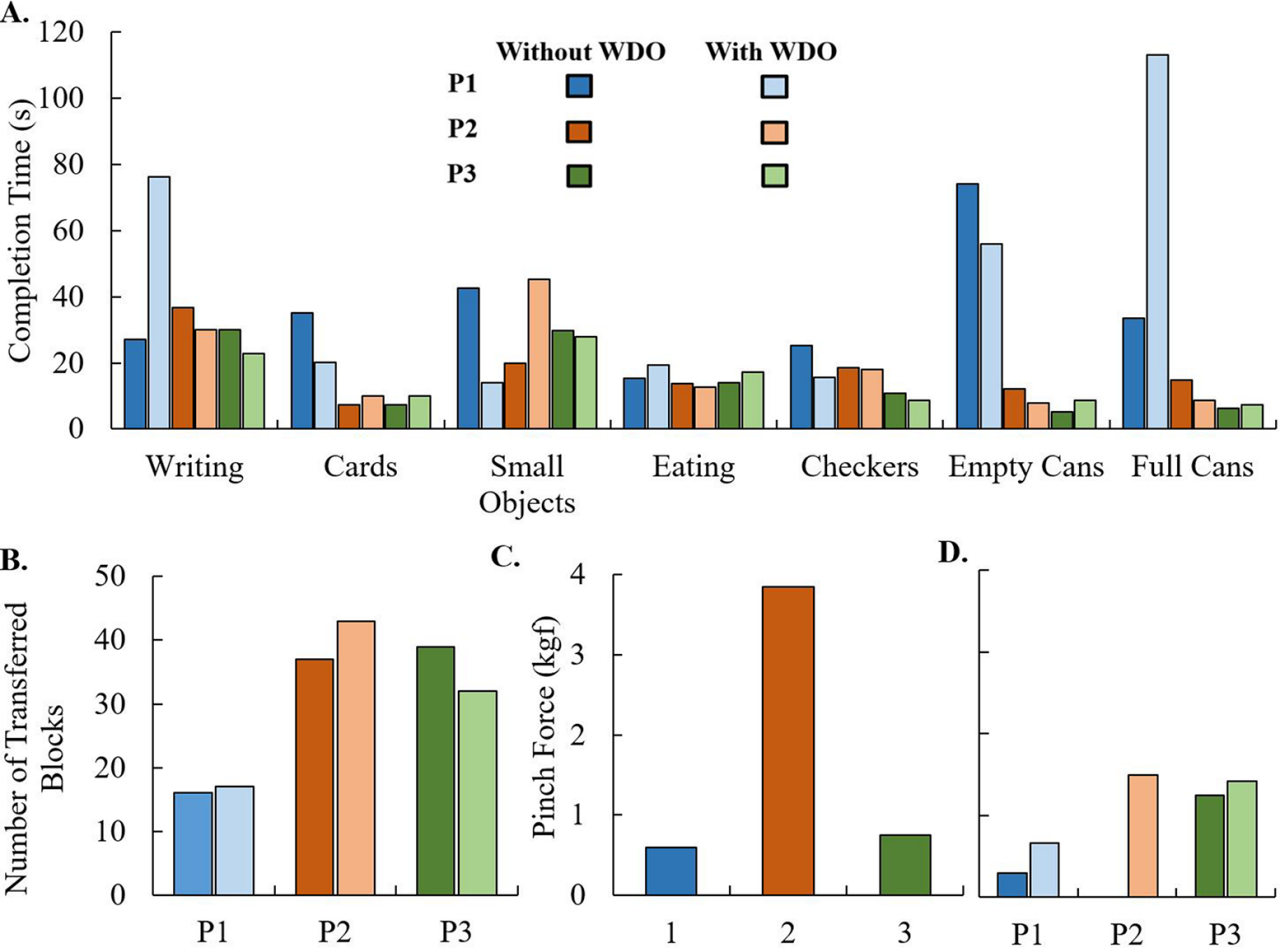
Users’ performance with and without 3D-printed WDO. (A) Performance during JTHF Test. (B) Performance during Box-and-Blocks Test. Users’ average pinch force during a (C) key grip without the WDO and (D) during a three-jaw chuck grasp with and without the WDO.

Testing with and without the 3D-printed WDO during the functionality tests demonstrated mixed improvements in hand function and ADLs (Fig 6). Two out of the three users exhibited improvement during the Box-and-Blocks Test with the 3D-printed WDO, managing to transfer more blocks under one minute with the WDO than without (Fig 6A). During the JTHF Test, we observed a greater variation in users’ abilities to perform tasks (Fig 6B). For example, while P1’s time to complete the small object task was improved by 28.6s with the WDO, P2 took 25.3s longer to complete the same task with the WDO (P1, P2, P3 are participants’ ID names, Table 2). Similarly, for the writing task, we observed improvement in completion time with the WDO by 7.4s for P3 whereas P1 took 49.3s longer to complete the same task under the same conditions. The total times to complete all tasks without the WDO were 252.5s, 122.6s, and 103.8s for P1, P2, and P3, respectively. With the WDO, these times were 314.4s, 132.6s, and 102.5s. When testing pinch force, two users (P1 and P3) were able to increase the strength of their three-jaw chuck grasp by 122.2% and 13.3% respectively, while P2 was able to perform this type of grasp for the first time (Fig 6C). All users noted that a key grip was more common and natural than the three-jaw chuck grasp without the WDO, but the WDO allowed them to perform the more common three-jaw chuck grasp.

**Fig 7:**
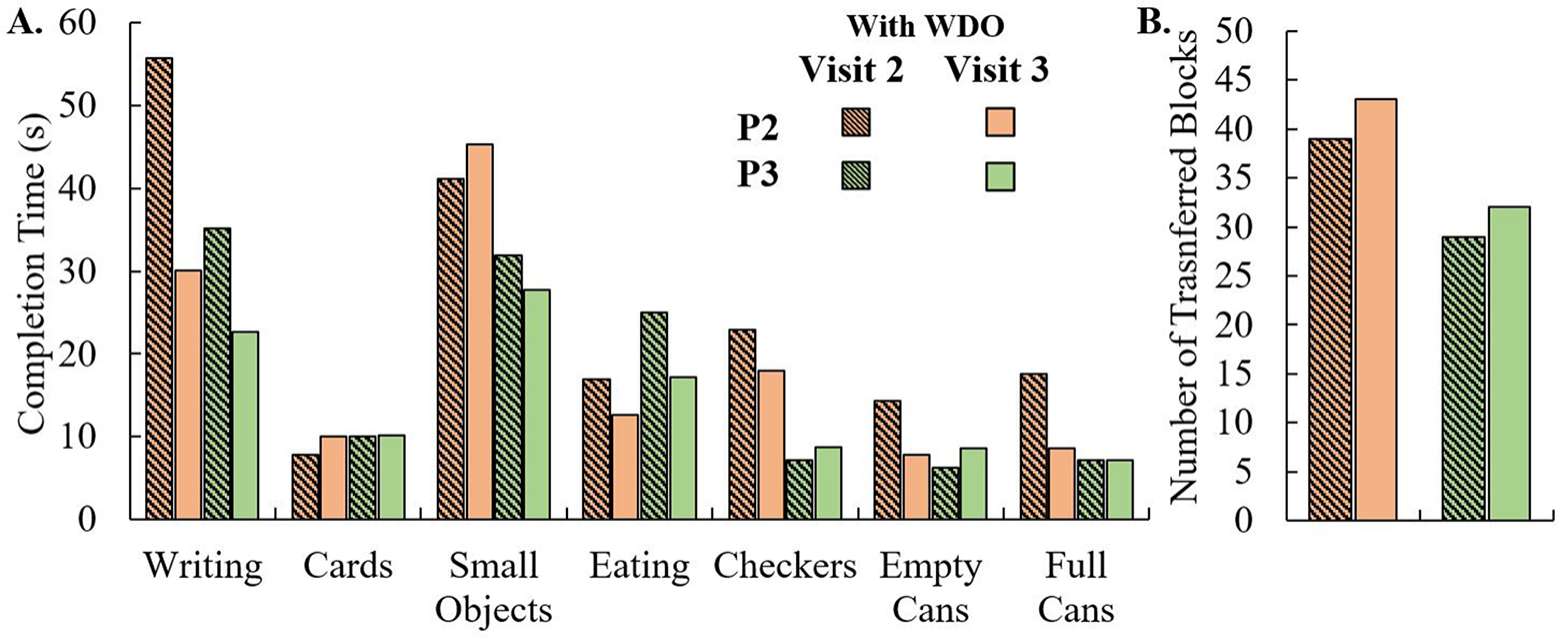
Comparison between visits 2 and 3 for P2 and P3. (A) JTHF Test. (B) Box-and-Blocks Test.

Both P2 and P3 participated in a follow-up visit (Visit 3), where they completed the same functionality tests as Visit 2 but with a WDO modified based on their feedback (Fig 7). Their results from the Box-and-Blocks Test (Fig 7A) and JTHF Test (Fig 7B) with the WDO were compared between Visits 2 and 3. Variability in performance during the JTHF test was observed during Visit 3 as well. The total time to complete the JTHF test decreased during Visit 3 by 44s and 20.3s for P2 and P3, respectively. Improvements in user performance between the visits were observed during the Box-and-Blocks Test. P2 and P3 transferred by 4 and 3 more blocks, respectively, with modified WDOs.

Users provided valuable feedback and positive impressions about the 3D-printed WDO (S1 Table). For example, one user noted that the “WDO makes the performed action a lot more duplicable [whereas] without the device, [he needed] to figure out a new way [to perform the same action] each time.” Another user suggested that using the 3D-printed WDO “would provide more precision and allow [her] to use different tools in the studio”.

## Discussion

Through this study, we developed a low-profile, lightweight, 3D-printed WDO that received high ratings on comfort, aesthetics, ease-of-fabrication, and customizability. A human-centered design approach was used during the development of the device with the aim to improve user acceptance, function during ADLs, and ease of fabrication and fitting. Results from testing with P&O students demonstrated a decrease in both the time and cost through 3D-printing, with positive comments and excitement for future use. Testing with users highlighted the ability of 3D-printing WDOs to improve performance during certain ADLs and to increase the strength of a three-jaw chuck grasp.

3D-printing hand orthoses shows promise as a cost-effective alternative to off-the-shelf and custom-fit metal devices. The 3D-printed WDO only required 1.5 hours of hands-on assembly time and is highly modular, with part replacement taking just a few minutes. In comparison to the laborious process of contouring metal for traditional WDOs, 3D-printing can potentially lead to more flexibility in the fabrication and repairing of hand orthoses and other similar devices. As a manufacturing process, it can be used in the medical field to improve device accessibility, availability, customizability, and, perhaps, acceptability by users.

During the functional tests, all of the users were able to perform an enhanced three-jaw chuck grasp with the WDO. The users also noted that the 3D-printed WDO allowed them to pick up objects in a more consistent way in comparison to free hand, for which they had to devise a new strategy for tasks each time. The assistance of the WDO was particularly useful during the Box-and-Blocks Test, resulting in improved performance for P1 and P2. The users also commented on increased ROM in the fingers with the WDO, which enabled them to grasp cans during the JTHF Test.

To encourage further testing and design improvements, all design files, manual, and instructional videos on device fabrication and assembly are available for open-source use and further development (WEBSITE, to be provided on publication). Other communities have seen the potential power and accelerated development provided by open-source designs and community-based development. For example, *Enabling the Future*, an open-source community for developing assistive devices for individuals with upper-limb loss, has grown to a community of over 7000 members in just four years. This community has accelerated development in hand prostheses mainly for underserved populations, for whom traditional devices are too expensive or unavailable (*e*.*g*., children) [50].

Making the WDO designs open-source will hopefully allow others to iterate on the designs based upon specific user needs. For example, a designer could modify this model for users with a specific functional impairment like thumb spasticity. In our testing, it was found that while the current option of the external thumb support was appropriate for users with a flaccid thumb, it was unsuitable for individuals with increased spasticity. For such a user group, an internal thumb support can be modeled to stop the thumb from flexing towards the palm (*i*.*e*., adducting) preventing the desired three-jaw chuck grasp. Our goal is that other engineers, clinicians, and users will be able to use these designs for continued open-source development. Open-source designs can drive collaborative innovation in the field of hand orthoses, laying ground between CAD enthusiasts and clinicians. In addition, these designs can potentially help introduce CAD modeling and additive-manufacturing techniques to P&O programs.

Despite its many advantages, open-source development also poses potential challenges for orthotic design. For example, for licensed orthotists, issues related to liability, longevity, and safety need to be addressed [51,52]. As a result, development needs to continue with multidisciplinary teams to ensure user-safety and long-term improvements in function. It is also important to understand the issue of potential over-prescription of devices that can arise from making certain technology open-source. Hence, the provision of such orthoses should be monitored closely by certified professionals.

In this study, a human-centered design approach was implemented in which WDO design was improved through iterative feedback from clinicians and users. In several other studies, user involvement has been shown to improve usability and acceptability of new devices [53,54]. In addition, usability tests can also be beneficial in identifying problems with existing devices [55]. In our study, we observed similar benefits, as the orthotists and individuals with SCI transitioned from “users” to “designers.” The impact of human-centered design was also seen through the improvement of average scores for the 3D-printed WDO as a result of incorporating feedback received during user testing. For example, after P1’s suggestion, Velcro attachments for hand straps were replaced with magnets, resulting in a range of affirmative comments from subsequent participants on the positive effect of the magnets for ease of donning and doffing.

While functionality is the primary goal of many assistive technologies, aesthetic appeal and comfort levels of a prescribed device are critical for many users and have a great impact on device acceptability [50]. Orthotic education commonly suggests that orthoses must be “aesthetically acceptable to the client,” “as lightweight and as simple as possible,” as well as “designed for easy adjustability” [56]. Positively affecting the functional outcomes for users, improved acceptability has a direct link to an increase in use of a prescribed device. The 3D-printed WDO designed in this study is lighter than traditional WDOs and offers greater aesthetic options where users can choose colors for individual components and straps. Users with prior experience with traditional WDOs noted that the 3D-printed WDO was more comfortable and aesthetically appealing than its metal version. In addition, the fabrication process of the developed 3D-printed WDO was highly modular, allowing for fast and inexpensive part replacement and repair. This has the potential to improve acceptance of WDOs by users who, in the past, might have worried about the costs and time associated with upkeep and maintenance.

The level of customization that 3D-printing provides can also be applied to the field of pediatric assistive technology, which often has less orthotic options than the adult market. Due to the limitations of current orthotic fabrication techniques, many orthoses, such as WDO, do not have a pediatric option as it may be challenging to fabricate smaller components. This leaves a portion of affected populations without necessary care and assistive technologies. As a result, 3D-printing can be a great solution to this problem, enabling fabrication of smaller parts. It can also result in devices much lighter than their traditional versions, making it a suitable option for children. Lastly, the ease of modifying color and design of a 3D-printed product can make devices more appealing to pediatric communities.

With the increase in the availability levels of 3D-printing technologies, many educational programs are integrating additive manufacturing into P&O curriculums. The instruction manual was created to assist with assembling instructions, but fundamentals of additive manufacturing tools will require on-going education and training for practitioners. There are also extensive on-line resources to support education and training for additive manufacturing. Integrating additive manufacturing processes into educational programs will continue to accelerate improvements in fabrication and design from these tools.

Limitations of this study include the functional heterogeneity of the user population, subjectivity of reported device scores, and lack of traditional WDOs for functional comparison. Our users included individuals with both complete and incomplete SCI, with varying injury locations that resulted in differences in hand functionality between users. Thus, while some users found the 3D-printed WDO to be useful for one task, others commented on it hindering their hand function during the same task. However, these inter-subject differences highlight the variability in user needs and function, underlining the need for customizable orthotic devices. Future studies with a larger set of users can help identify the different design features that enhance function for specific users.

Subjectivity of reported device scores and user comments is an obvious limitation of this study. An unbiased evaluation of the device was attempted to be collected through mixed-methods approach that included surveys, functional testing, and interview questions. In addition, the majority of our participants were very excited over the project idea, providing positive comments about the developed device and the fabrication technique, which might explain the high scores and positive comments given to the device by the participants. In future studies, larger participant population with mixed background and comfort levels with new technologies or advanced manufacturing may produce more comprehensive results and opinions.

Our users were also highly practiced and very dexterous without using a WDO, with an average of 20.8 years since injury. Furthermore, they were testing the 3D-printed WDOs for the first time with about 10 minutes of acclimatization time. Our users pointed out that development of a tenodesis grasp, which a WDO mimics and attempts to strengthen, is an essential part of the rehabilitation for individuals with cervical SCI. Many individuals with SCI are initially prescribed a WDO and then transition to performing daily tasks without the device as they develop their tenodesis grasp. Our users noted that the 3D-printed WDO may have been particularly useful in the first years after their injury when they were regaining strength and working on rehabilitation. Two users in our study had been prescribed a WDO in the past, but stopped using them and no longer had the WDO. Thus, a comparison with custom-made traditional metal WDOs was not possible in this research. Future studies that (1) compare multiple alternative design of WDOs, including both 3D-printed and traditional designs, (2) allow greater time for practice with the devices, and (3) longitudinally evaluate function and performance after use in daily life can further improve the evaluation and design of WDOs and other body-driven orthoses.

While 3D-printing provides a new toolset for rapid prototyping and manufacturing, testing of material properties and durability of 3D-printed devices has been limited [57–59]. Many P&O students expressed particular concern about the durability of the 3D-printed WDO during the study. As a result, fatigue testing of devices should be conducted to determine the durability of 3D-printed products for daily use and to identify locations of failure after extended use for design improvements. In this study, we used conservative printing properties to improve the durability of devices. However, further research on material properties can help optimize the weight, profile, and durability of 3D-printed assistive technology. In addition, laser sintering techniques or other form of additive manufacturing may also be implemented to fabricate a more durable WDO.

## Conclusions

In this study, a novel fabrication approach involving additive manufacturing techniques and CAD modeling was utilized to produce WDOs for individuals with SCI. The model of the 3D-printed WDO was developed to mimic the traditionally prescribed metal hand orthoses and improvements in the design and functionality of the device were added. These improvements were made based on the feedback received from testing with the P&O students and WDO users in a human-centered design approach. Changes in the fabrication costs and time with the use of 3D-printing technology were also quantified. Introducing additive manufacturing to the fabrication of traditional hand orthoses was able to reduce both the costs and time of the production of each device and allowed for more customization requested by either an orthotist or user. The 3D-printed WDO also demonstrated some improvements in hand function during certain ADLs and received positive feedback on scores for function, aesthetics, comfort, ease of donning, and suitability for needs for the tested users.

Integrating additive manufacturing techniques with human-centered design approaches can stimulate innovation and improve the quality of life for individuals with SCI and other disabilities. Our experience has demonstrated that 3D-printing has the potential to increase the accessibility of orthotic solutions (*i*.*e*., WDOs), decrease the time clinicians spend fabricating orthoses, and improve function on ADLs for individuals with SCI. In addition to increasing availability of comfortable and aesthetically-appealing orthoses for adults, 3D-printing may provide orthotic solutions to pediatric populations and other groups with limited options. These additive manufacturing techniques also have the potential to improve access to orthotic solutions for individuals with SCI in other countries or low-resource settings. Besides encouraging further development in WDO designs, the investigation team hopes that this open-source model will provide new grounds for testing other orthotic devices that can enhance motor learning, rehabilitation, and quality of life for individuals with SCI.

## Supporting information

S1 Manual The final version of the device manual used to properly size, fabricate, and assemble a right-hand 3D-printed WDO. (PDF)

S1 Survey The example of a survey provided to P&O students after they completed the assembly of a 3D-printed WDO. (JPG)

S1 Video One-minute video demonstrating users hand function with and without the 3D-printed WDO. (MP4)

S1 Table Comments from the P&O students and the user groups made in regard to the 3D-printed WDO. (JPG)
