# Supplementary material for: Design of a 3D-printed, open-source wrist-driven orthosis for individuals with spinal cord injury
Source: PLoS One. 2018 Feb 22;13(2):e0193106. doi: 10.1371/journal.pone.0193106 (PMC5823450; doi:10.1371/journal.pone.0193106)
Supplement: S1 Manual — (PDF) [file pone.0193106.s001.pdf]

3D-Printed Wrist-Driven Orthosis Manual (Right Hand)

Ability & Innovation Lab
University of Washington, Seattle

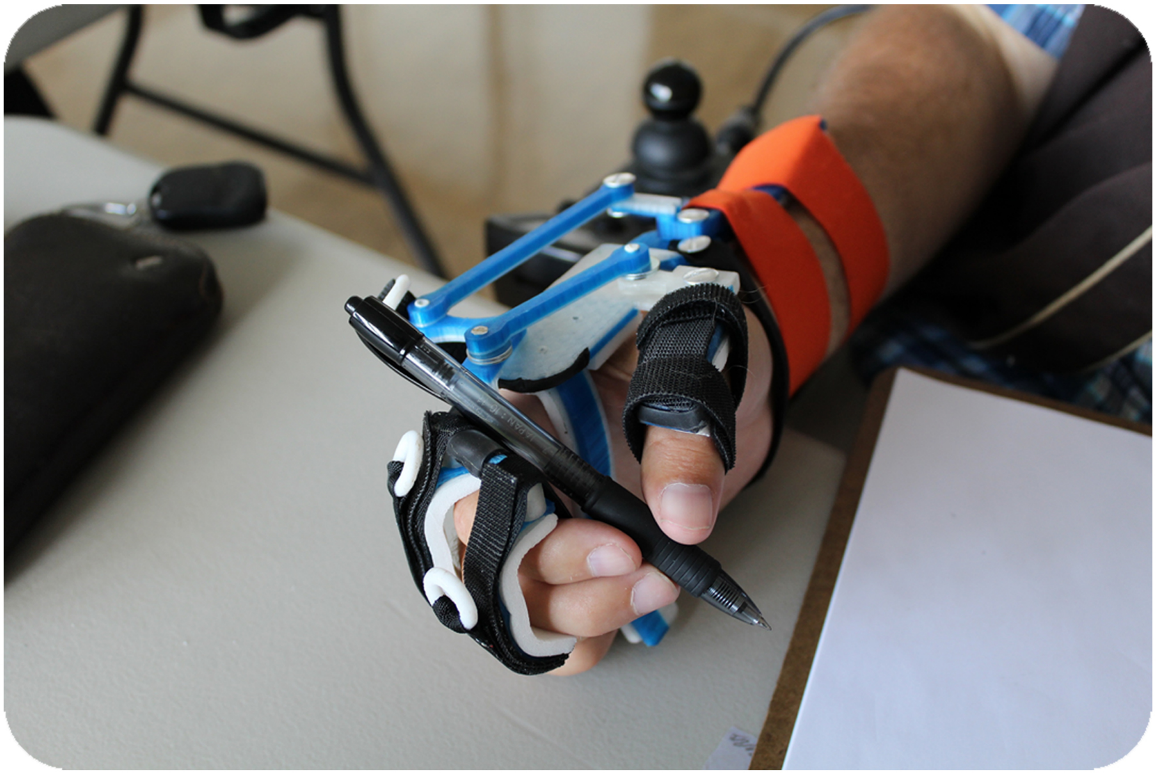

Table of Contents

1. Introduction and Description 1
    1.1 General Recommendations 2
    1.2 Acceptance 3
2. Determining Size and Saving as STL 4
    2.1 For Users without CAD Experience 4
    2.2 For Users with CAD Experience 7
3. Printing Specifications 11
4. Additional Materials and Tools 13
5. Assembly 17
    5.1 Support and Raft Removal 17
    5.2 Padding, Velcro, and Strap Preparation 18
    5.3 Soldering ABS Pins 20
    5.4 Reference Page and WDO Assembly 23
    5.5 Post-Assembly 28

Introduction and Description

This 3D-printed wrist-driven orthosis (WDO) is commonly prescribed for individuals with spinal cord injury, who lack mobility in their fingers but can move their wrists. This device facilitates a pinching grasp with wrist flexion and extension. This design is compatible with any version of the MakerBot MakerWare software and any of the FDM 3D-printers. This design was developed using SolidWorks 2014 and can be edited with any CAD-modeling software that can read ***.prt** or ***.sldprt** formats.

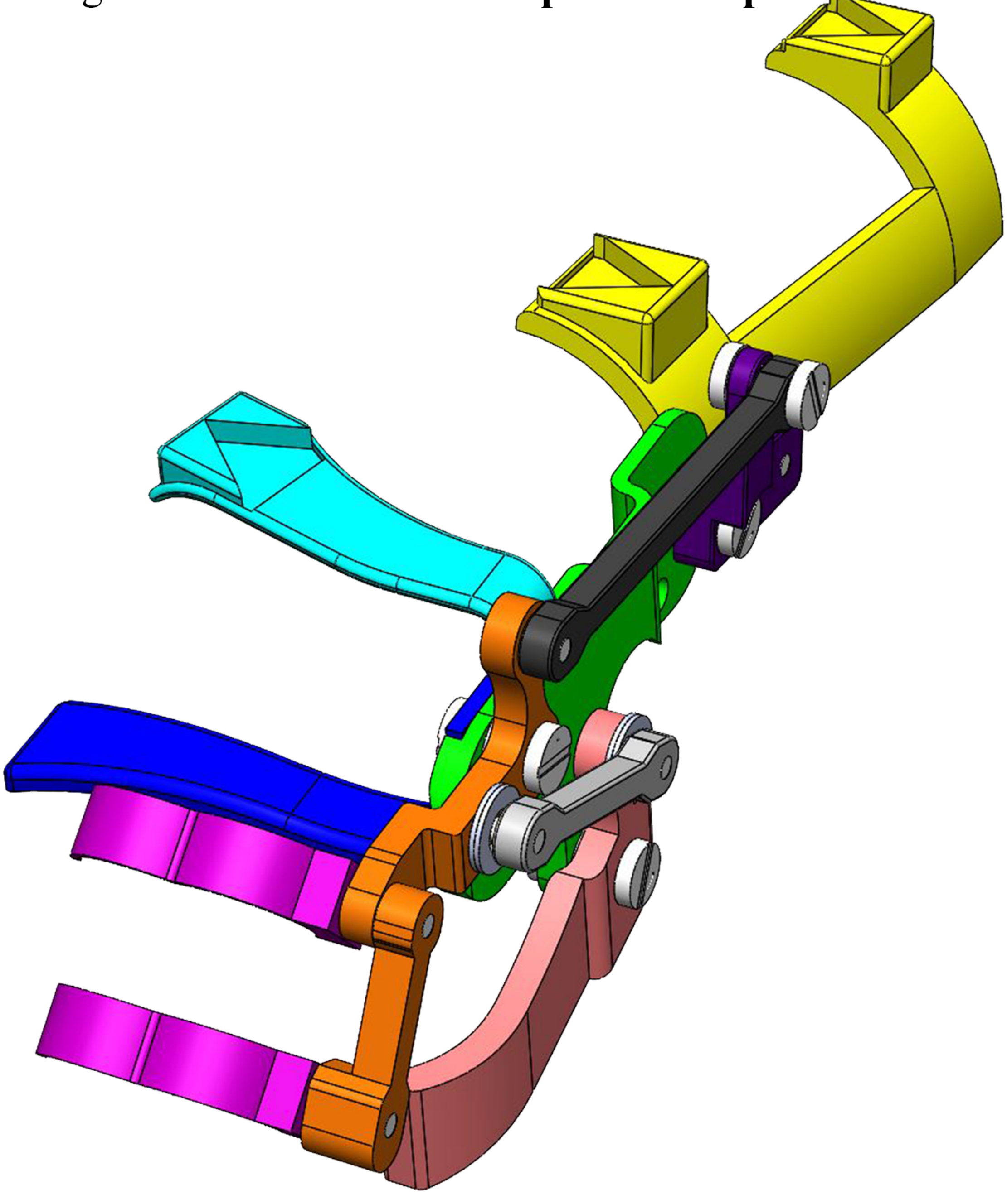

1.1 General Recommendations

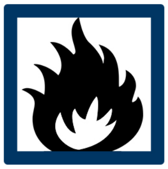

Do not expose the WDO to hot conditions (over 120° F) or fire because they are made out of plastic material (PLA) with a low melting point.

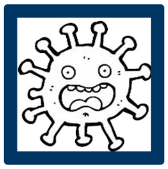

Ensure there are no areas that may cause pressure or skin irritation as it may lead to skin breakdown and poor hygiene.

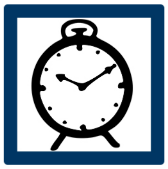

Check every 30 minutes for redness or skin irritation when first wearing the wrist orthosis. If you find redness or skin irritation, please stop using the wrist orthosis.

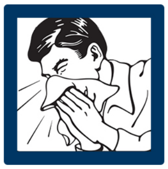

Ensure that you are not allergic to the printing material or any other components before assembling and wearing the device.

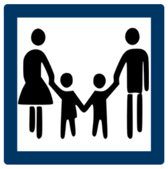

Children using this device must be supervised at all times.

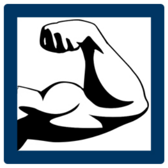

Begin using the WDO in a progressive manner. If the wrist muscles are fatigued, discontinue use until your muscles are back to full strength.

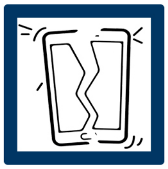

If any component of the WDO breaks or fractures, please stop using it immediately.

1.2 Acceptance

By accepting and downloading any files, design, plan, component, or assembly instructions related to this WDO, I understand and agree that any such information or material furnished by any individual associated with the design team is furnished as a gift for the sole purpose of evaluating various design iterations, ideas and modifications. I understand that such improvements are intended to benefit individuals having specific disabilities and are not intended for commercial use. I further understand and agree that there are no warranties expressed or implied associated with the provision of any such information or material.

Determining Size and Saving as STL

2.1 For Users without CAD Experience

1. Take the necessary measurements (in inches) of user's hand and wrist widths using the figures below.

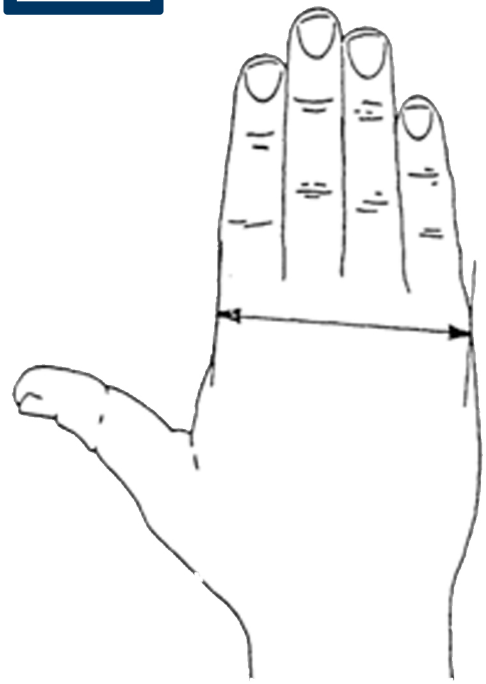

Figure 1. Hand width

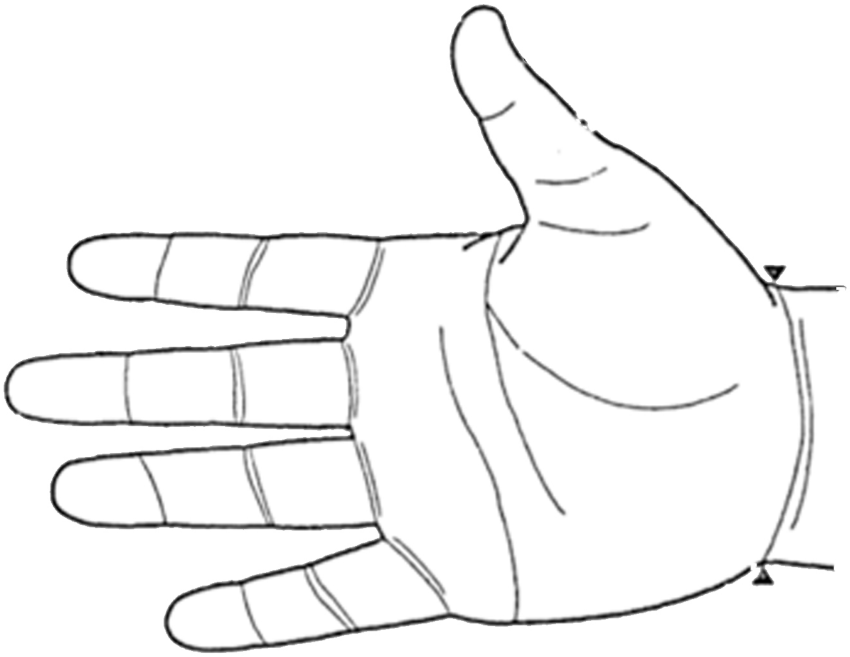

Figure 2. Wrist width

2. Look at the sizing chart below to determine sizes for device components. ***Note:*** if you find that your measurements do not fall into any of the sizing categories, you need to modify the WDO components separately.

	XS	S	M	L	XL
**Hand Width Dimension (Figure 1) (in)**	2.8-3.0	3.0-3.2	3.2-3.4	3.4-3.65	3.65-3.85
**Wrist Width Dimension (Figure 2) (in)**	1.95-2.15	2.15-2.3	2.3-2.5	2.5-2.7	2.7-2.9

Determined sizes correlate with the sizes of the following pieces:

HAND WIDTH

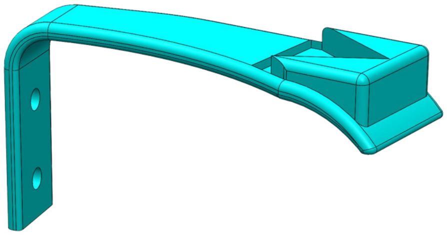

dorsal piece

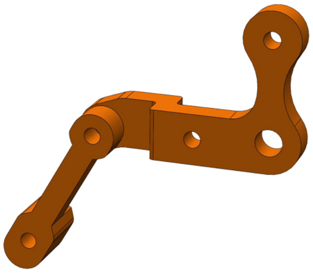

finger piece

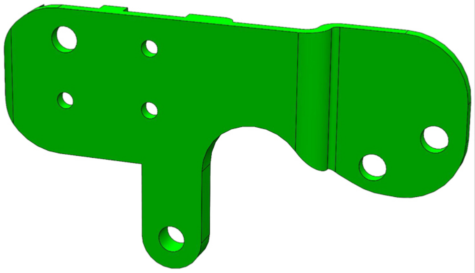

hand piece

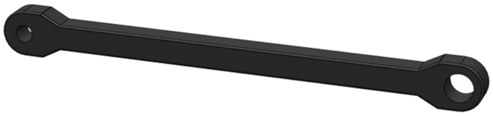

long bar

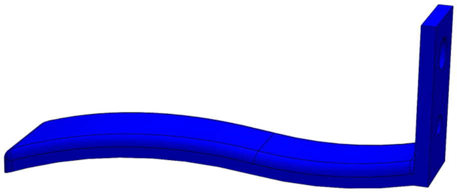

palmar piece

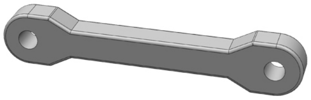

short bar

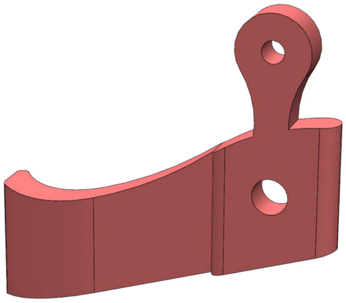

thumb piece

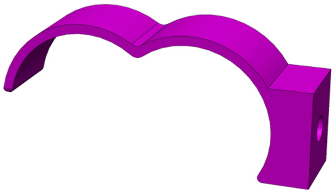

finger ring

WRIST WIDTH

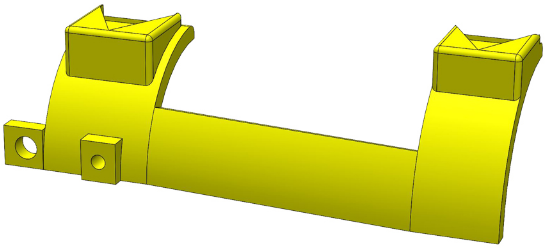

forearm piece

The following piece comes in one size:

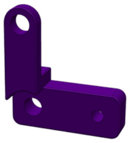

input link

3. Open each desired part in SolidWorks.

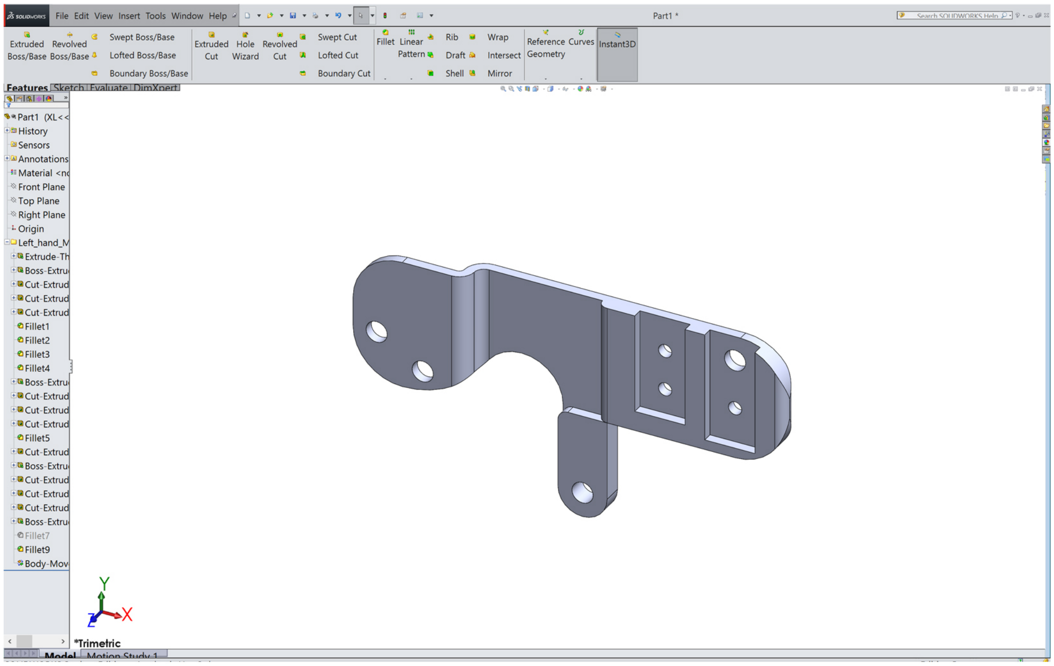

4. On the left-side panel, click on *Configuration Manager* (red box below) and double- click on the desired size from the list.

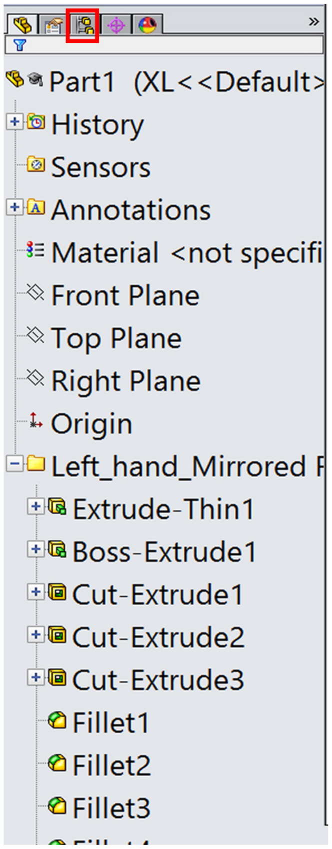

5. Now save the part in .stl format. Go to *File - Save As...* and choose *STL* under the *Save as Type.*

2.2 For Users with CAD Experience

(skip if already completed 2.1)

Take necessary measurements of user's hand using the figure below. We suggest taking measurements in milimeters (mm) and then converting them to inches (in).

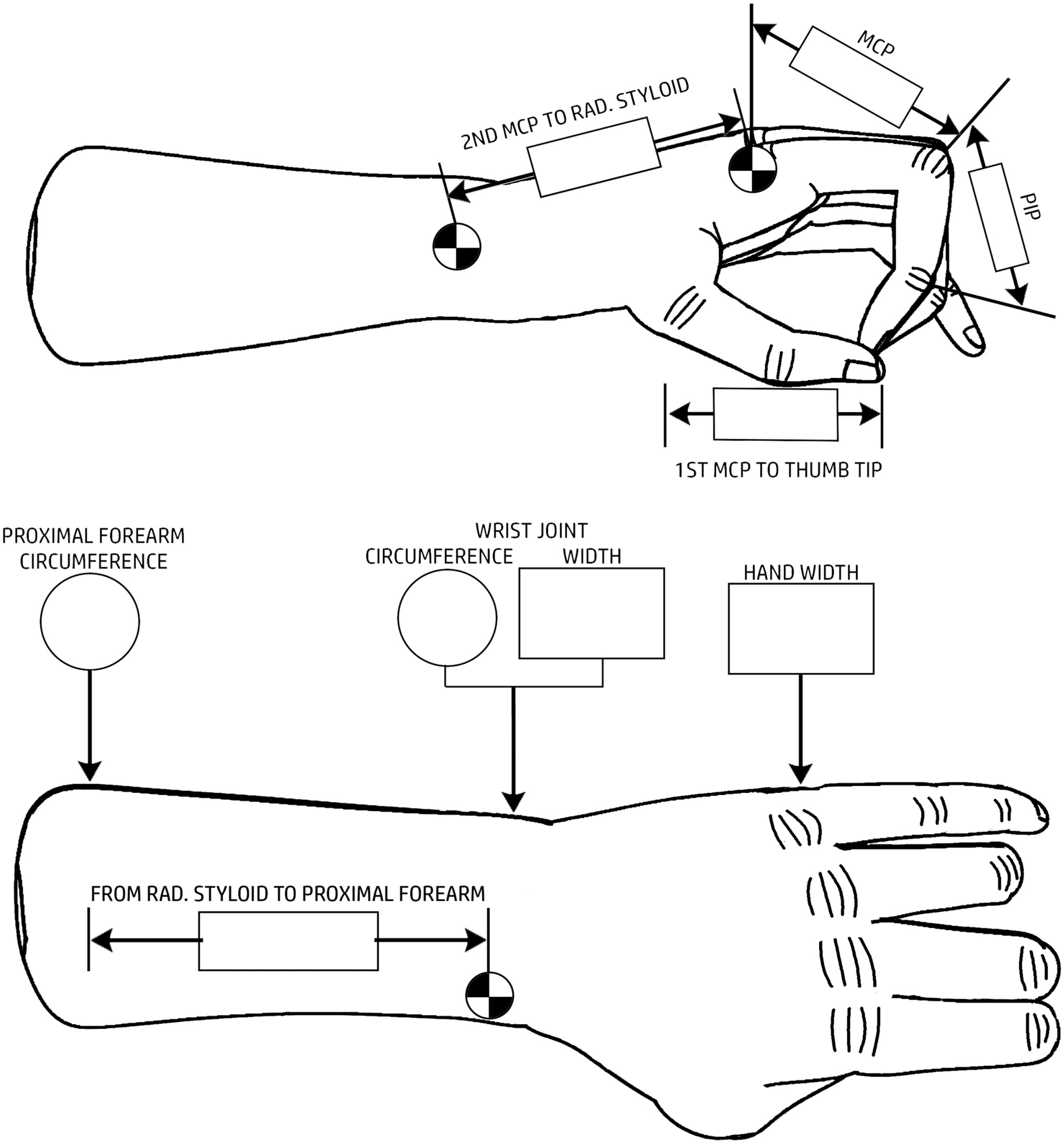

Use the following instructions to manually change component dimensions in accordance to the sizes obtained above.

1) Use the instructions detailed in 2.1 to determine and set the size for the following components: **hand piece, forearm piece, dorsal piece, short bar, finger ring.**

2) **Palmar piece**

a. Open the component file in SolidWorks and click on *Configuration Manager* from the left-hand menu. Right-click on *Design Table* and choose *Edit Table in New Window*

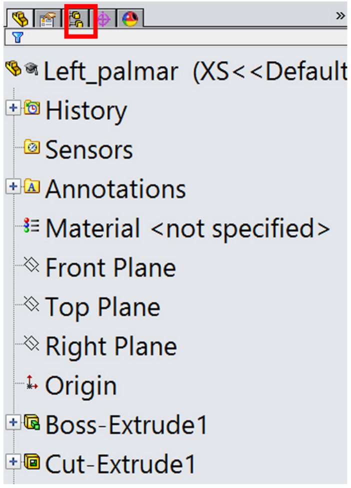

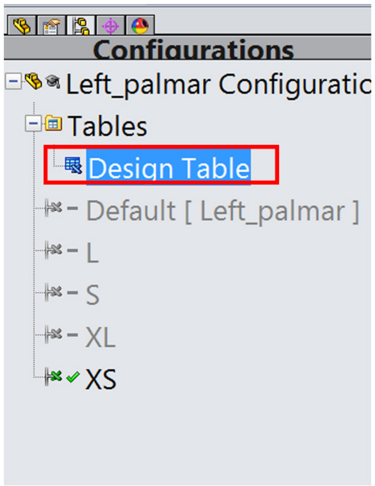

b. Look for the *D5@Sketch4* column. Choose the size that has the dimension in this column closest to the hand width dimension you measured before. Make a note of that size and close the table. **Do not make any changes in the table!**

c. Double-click on the size configuration determined above. Go back to *Design Tree* and double-click on *Boss-Extrude2*.

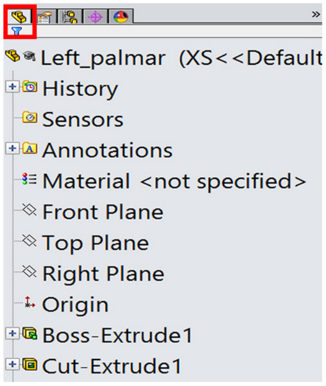

d. Look for the dimension named *D5@Sketch4* and double-click on it. Change it to the hand width dimenion (inches) and hit *Enter*.

e. Save the file in STL format as described in section 2.1.

3) **Long bar**

a. Open the component file in SolidWorks. Double-click on *Boss-Extrude1* from the design tree, look for the dimension named *D1@Sketch*1, and double-click on it

b. Change it to the dimension from the radial styloid to 2nd MCP (inches) and hit *Enter.*

c. Save the file in STL format as described in section 2.1.

4) **Thumb piece**

a. Open the component file in SolidWorks. Double-click on *Boss-Extrude1* from the design tree, look for the dimension named *D2@Sketch2*, and double-click on it.

b. Change it to the following: sqrt([1st MCP to thumb tip + 0.4]^2 - 0.75^2) (inches).

c. Save the file in STL format as described in section 2.1.

5) **Finger piece**

a. Open the finger assembly. It consists of two components. Left-click on *finger_side* and click *Open part*. Now you will be able to individually modify each component.

b. Click on *Configuration Manager* from the left-hand menu. Right-click on *Design Tabl*e and choose *Edit Table in New Window* (as described in 2a)

c. Calculate X = MCP - 0.19 (inches). Look for the *D6@Sketch1* column. Choose the size that has the dimension in this column closest to the calculated X value. Make a note of that size and close the table. **Do not make any changes in the table!**

d. Close the file and return to the asembly. Left-click on the *finger_side* and choose the appropriate size configuration from the list.

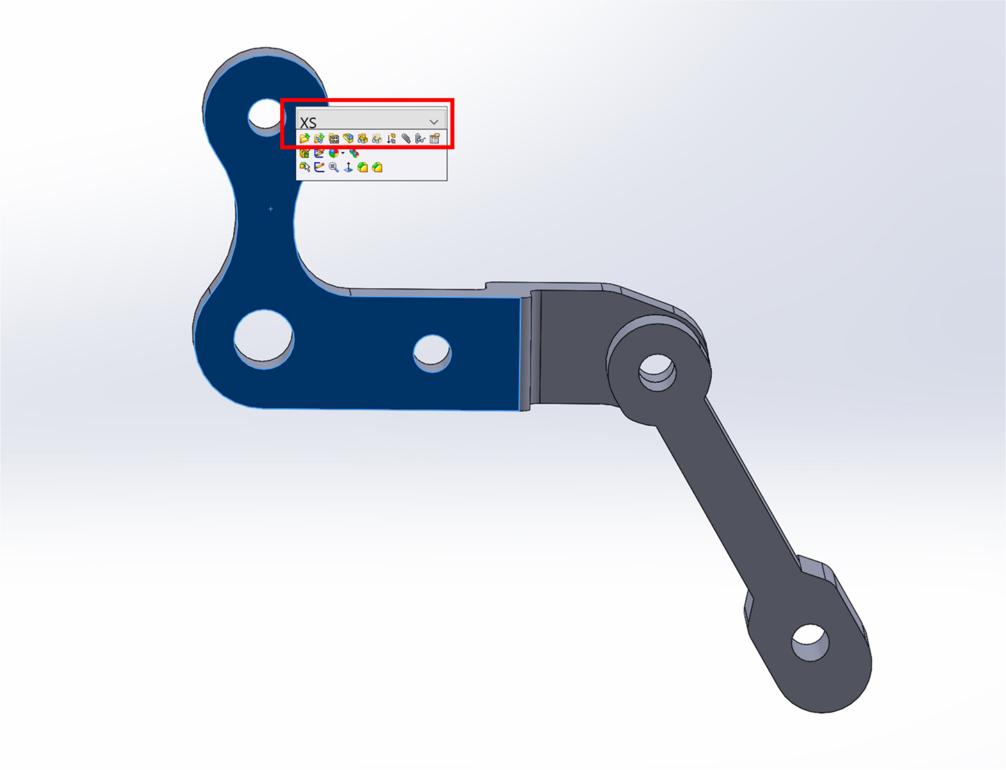

e. Left-click on *index_bar* and click *Open part*.

f. Calculate Y = PIP +0.51 (inches). Double-click on *Boss-Extrude2* and look for the dimension named *D2@Sketch2*. Double-click on that dimension and change it to the calculated Y value.

g. Now go back to the assembly file and hit *ctrl+B*. This will rebuild the file.

h. Save the file as a part file (**.prt, .sldprt**). It is important to save it first as a single part file. Now open that part file and save it as STL as shown in section 2.1.

Printing Specifications

1. Open MakerBot Desktop software. Specifications for 3D-printing components are as follows:

- ☐ PLA Filament
- ☐ Number of Shells: 4
- ☐ Extruder Temperature: 230°C
- ☐ Speed While Extruding: 60mm/s
- ☐ Supports: yes
- ☐ Infill: 30%
- ☐ Layer Height: 0.20mm
- ☐ Build Plate Temperature: 40°C
- ☐ Speed While Traveling: 90mm/s
- ☐ Rafts: yes

2. We recommend orienting parts on the buildplate in the way, shown in the figures below:

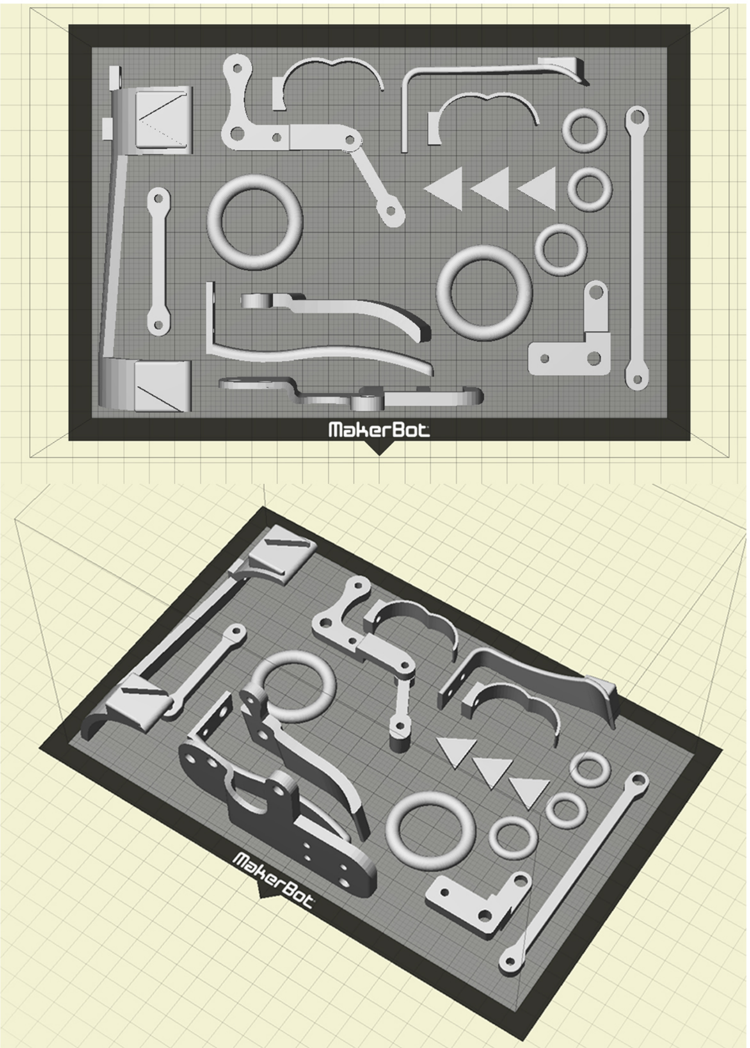

3. Use the following table to ensure that you have all necessary parts ready for printing:

- ☐ 1x dorsal piece
- ☐ 1x hand piece
- ☐ 1x short bar
- ☐ 1x finger piece
- ☐ 2x finger ring
- ☐ 2x ring_forearm
- ☐ 1x ring_hand
- ☐ 1x palmar piece
- ☐ 1x long bar
- ☐ 1x input link
- ☐ 1x thumb
- ☐ 1x forearm piece
- ☐ 3x triangle

4. Load the filament into the 3D printer and start the print.

Additional Materials & Tools

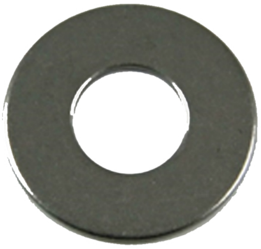

Washers
#10, zinc or aluminum, by Everbilt.
http://tinyurl.com/pcc2sep

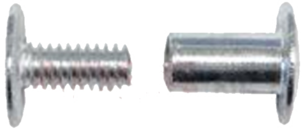

Aluminum Post-Screws
(A) ¼” long, 3/16” in diameter aluminum post-screws. Screw thread is #8-32. *http://tinyurl.com/zkxg7sq*
(B) ⅜” long, 3/16” in diameter aluminum post screw. Screw thread is #8-32. *http://tinyurl.com/gvqb6pw*

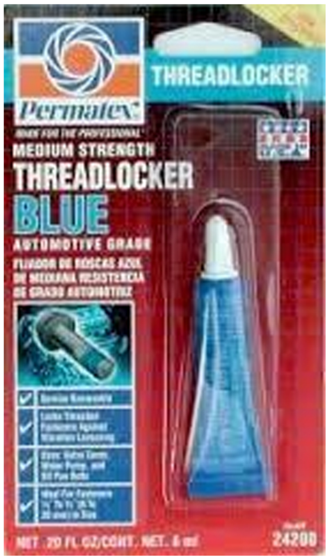

Screw Adhesive
A medium-strength thread locker is recommended.
http://tinyurl.com/nfjjr3c

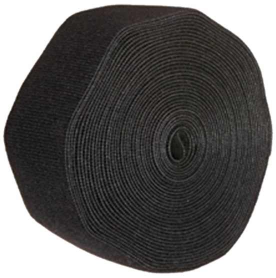

Self-Adhesive Velcro
It will be necessary to cut this to size.
http://tinyurl.com/htb6cwx

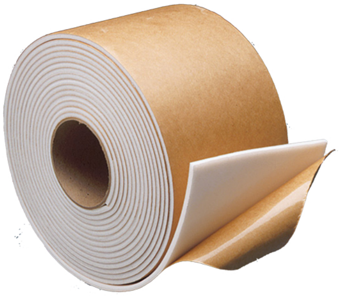

Foam Padding
Sold in a 25’ roll, will need to be cut to size.
http://tinyurl.com/m7dcm9y

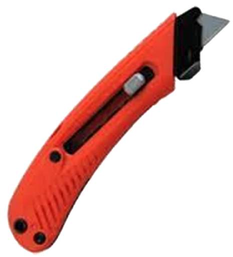

Scissors, Exacto Knife, or Box Cutters

Can be found at most hardware stores such as Lowe's, Ace, and Home Depot.

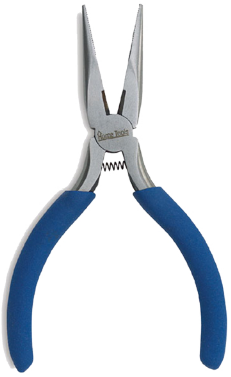

Pliers

Necessary for removing supports from printed components. Can be found at most hardware stores such as Lowe's, Ace, and Home Depot.

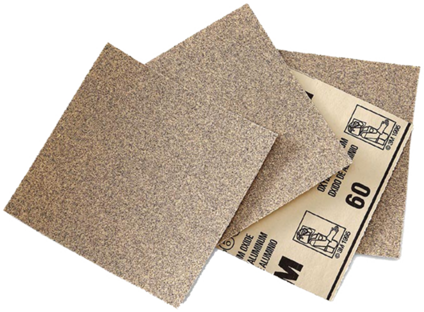

Sand Paper

The recommended range of grit number is 40-120. The grit number goes from rough to fine. Rough sandpaper will remove unwanted material faster whereas fine sandpaper will give better surface finish.

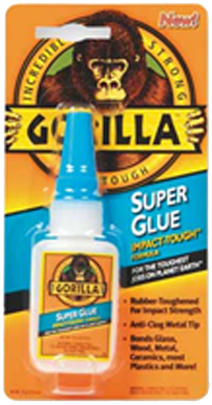

Superglue

Necessary for gluing Velcro straps to the printed components.

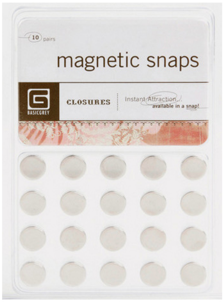

Self-Adhesive Magnets

1/32" thickness, approximately 3/8" in diameters with self-adhesive backing.
http://tinyurl.com/zl2mth6

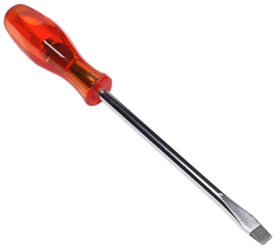

Screwdriver

Necessary for tightening the post-screws. Can be found at most hardware stores such as Lowe's, Ace, and Home Depot.

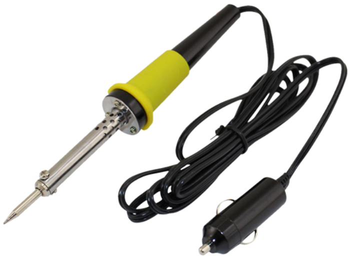

Soldering Iron

Necessary for melting ABS pins. Ensure that you have proper ventilation since breathing ABS fumes can be dangerous. A lighter can be used instead to carefully heat a large nail head.

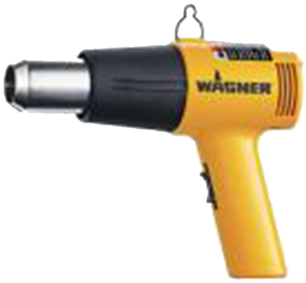

Heat Gun

Used to soften the thermoplastic part of the finger piece. Can be found at most hardware stores such as Lowe's, Ace, and Home Depot.

InstaMorph®
Moldable Plastic

Instamorph or Other Formable Thermoplastic

Necessary for creating a plastic shell around fingers.
http://tinyurl.com/hasfjfb

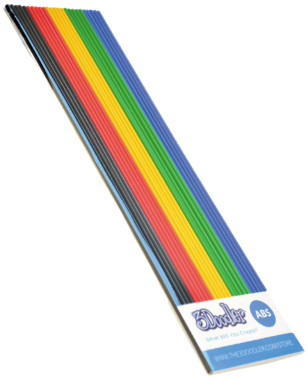

3mm ABS Strands

Used for creating ABS pins needed for assembly of the WDO.
http://tinyurl.com/j2fcxfk

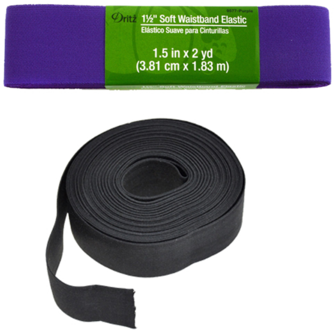

Elastic Straps

Necessary to secure the WDO on user's hand. We recommend 1.5" width.
http://tinyurl.com/h4bx7nl

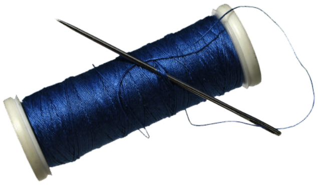

Thread & Needle

We recommend using an industrial-strength thread and a thicker needle.

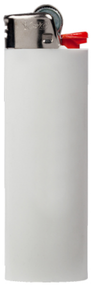

Lighter

Used to slightly burn cut edges of elastic straps. Can also be used for creating ABS pins.

Assembly

5.1 Support & Raft Removal

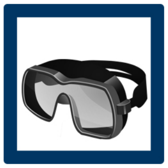

1. Safety first! Put on your goggles before removing any supports and rafts.

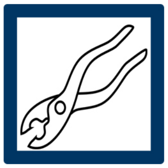

2. Begin with removing rafts with pliers. We recommend gently twisting on rafts around the part. Do not overbend it. Usually, rafts can be removed in one piece.

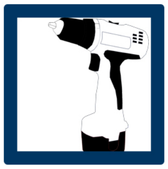

3. Remove supports with pliers and wire cutters. Be careful, applying too much force might break some pieces. We recommend drilling through holes a few times. Use 1/8” drill for small holes and 3/16” for larger holes.

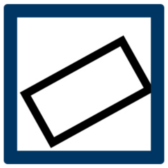

4. Use sand paper to sand rough edges and faces until smooth. Soldering gun can also be used to smooth out WDO components with rough surfaces. **Be careful not to burn yourself.**

5.2 Padding, Velcro, & Strap Preparation

1. Cut strips of padding for the following pieces: *forearm, hand, dorsal, palmar, thumb*. The padding will attach on the inner side of each part where user's skin is in contact with the device.

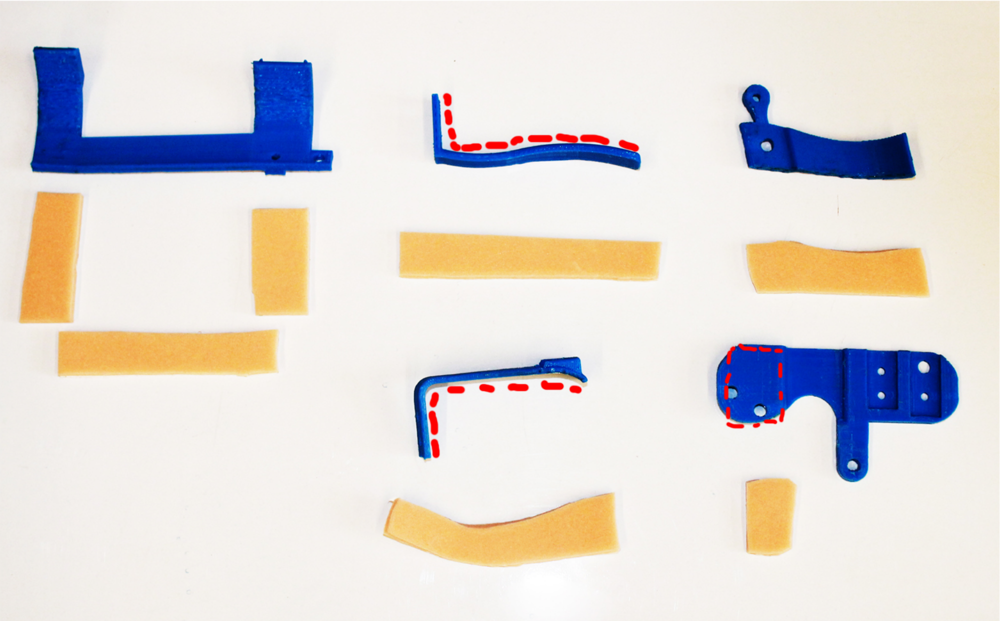

2. Using 1.5" wide elastic waistband, cut two forearm straps. The length of each strap is determined by:

- proximal strap: proximal forearm circumference (mm) + 110mm
- wrist strap: wrist circumference (mm) + 110mm

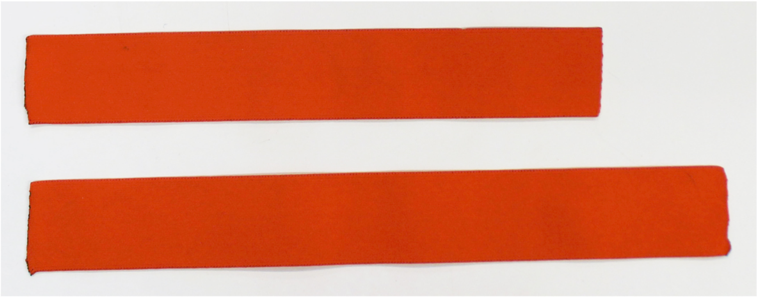

3. Using a lighter, carefully burn off the cut edges.

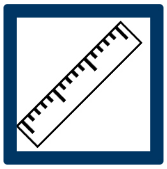

4. Wrap a measuring tape from the radial styloid to the middle of the back of the hand (third MCP) under the palm (as shown below). Then add 20mm to that measurment - this will be the length of the hand strap.

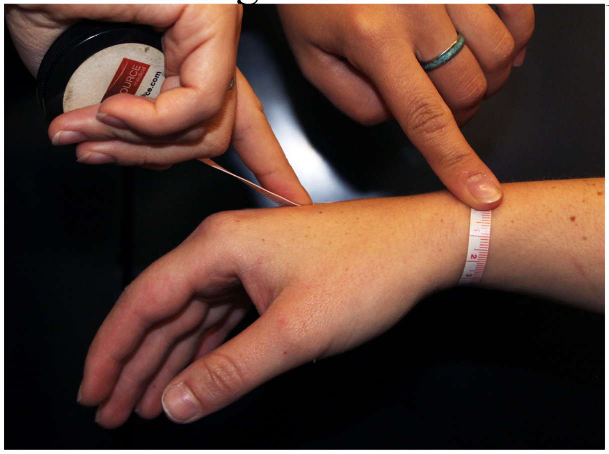

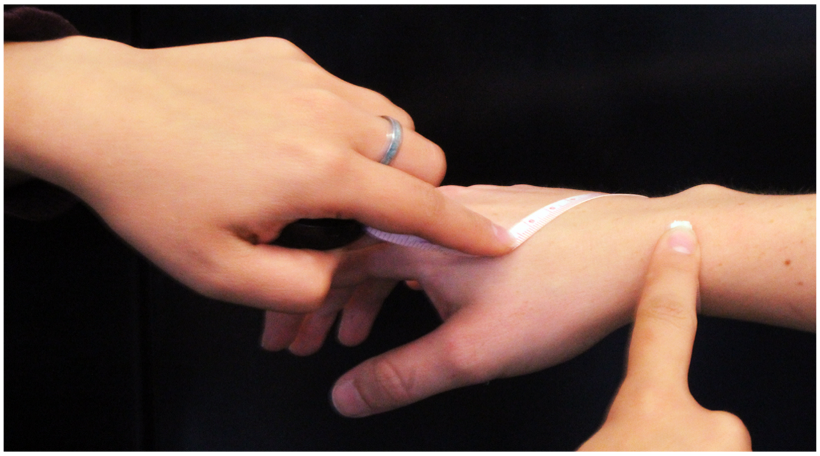

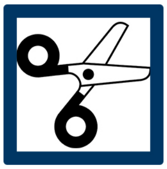

5. Cut a 16mm wide hand strap using an elastic waistband and burn the cut edges with a lighter.

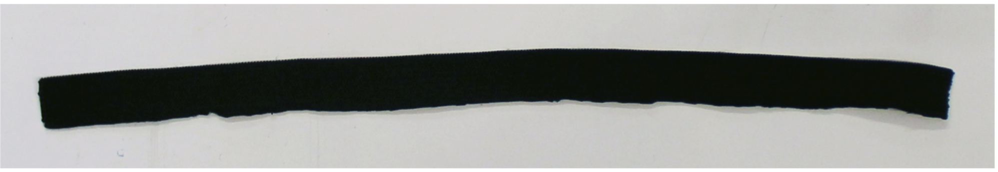

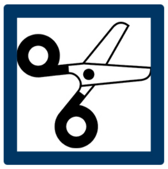

6. Cut a 25mm wide, 115mm long strap for the thumb using an elastic waistband and sligthtly burn the cut edges with a lighter. Cut a soft side of self-adhesive Velcro with the same dimensions.

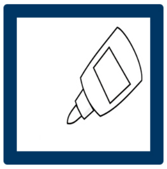

7. Use superglue to securely attach the soft Velcro piece to the thumb strap.

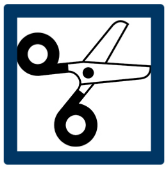

8. Cut a rough side of self-adhesive Velcro to cover the outter side of the thump piece.

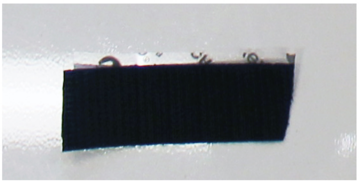

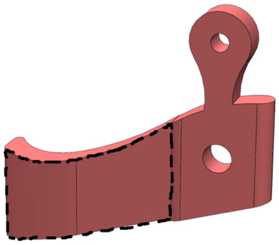

5.3 Soldering ABS Pins

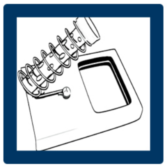

1. Prepare soldering gun, fan, wet sponge, pad for cleaning the gun tip, ABS pins, and wire cutters. Make sure the work area is well ventilated since breathing ABS fumes directly can be dangerous.

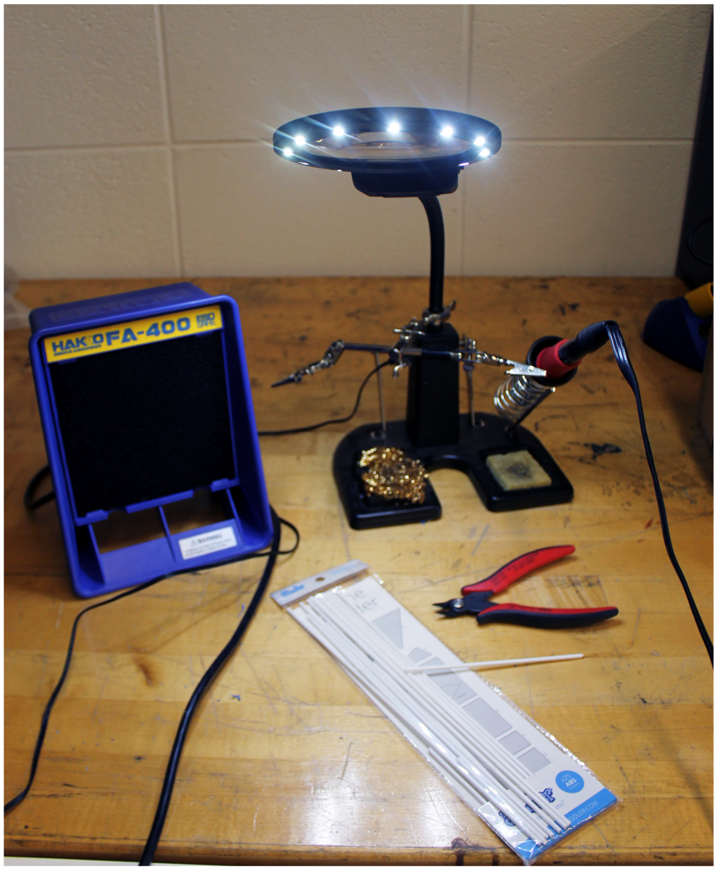

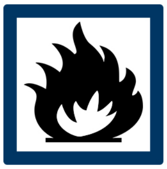

2. Melt the tip of the ABS pin. **Be careful not to burn yourself.**

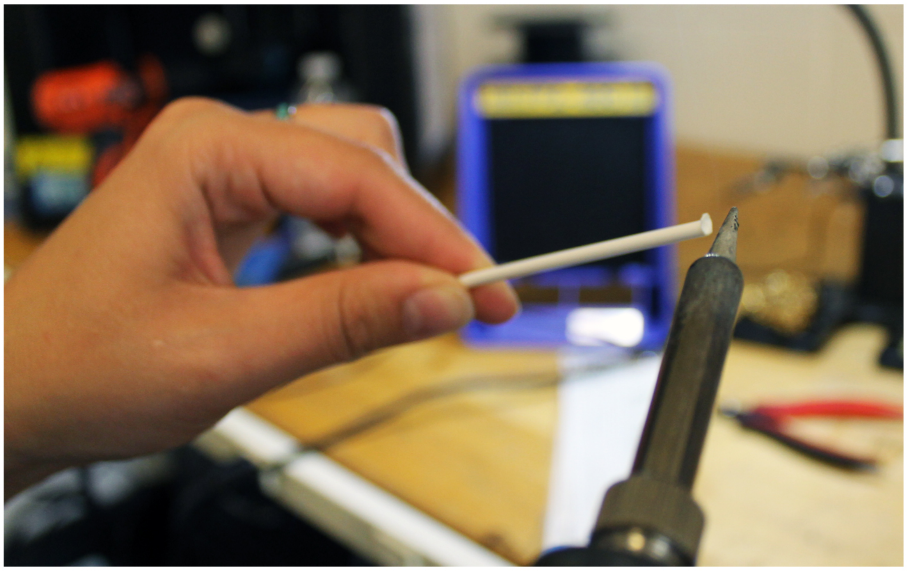

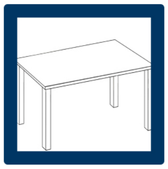

3. Place the melted tip plat against the table and hold it for a few seconds.

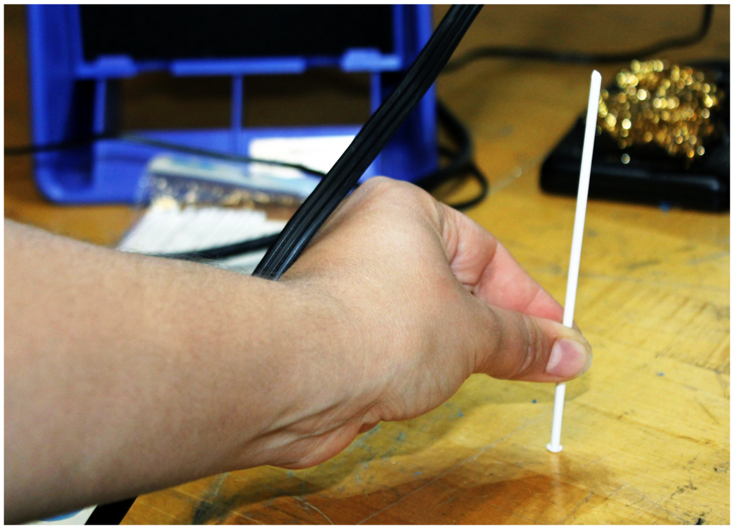

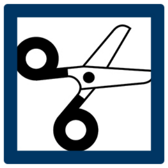

4. Insert the pin through the holes and, using wirecutters, cut the other end of the pin, leaving a small tip.

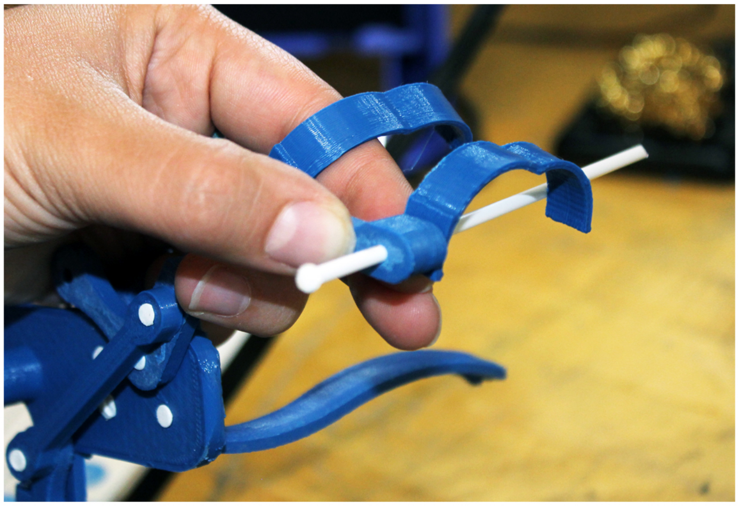

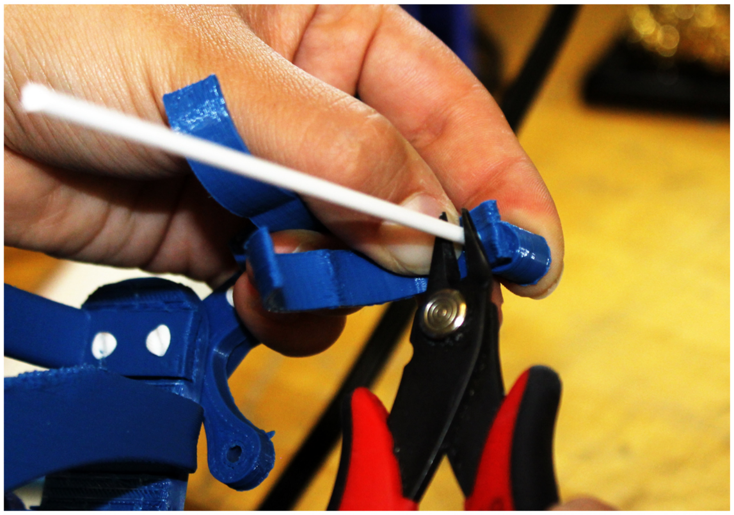

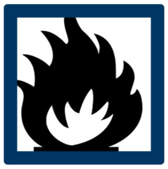

5. Melt the remaining end of the pin and press it against the component surface.

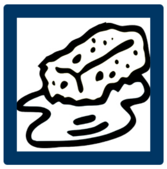

6. Don't forget to clean the soldering gun tip after every use.

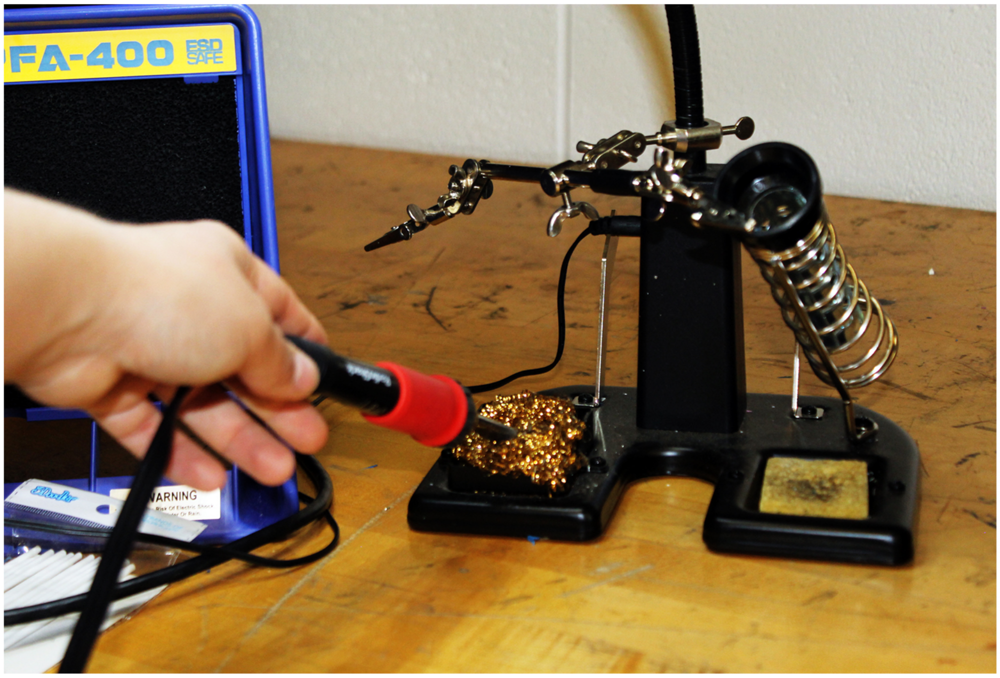

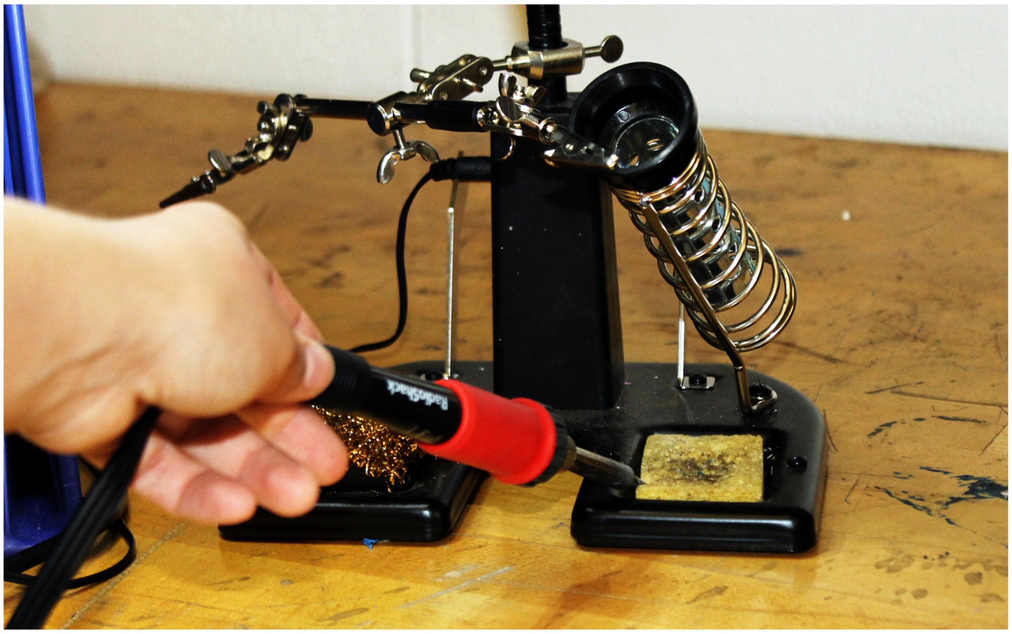

5.4 Reference Page & WDO Assembly

1x

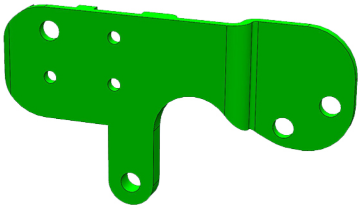

1x

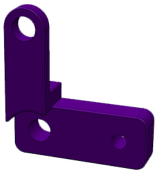

1x

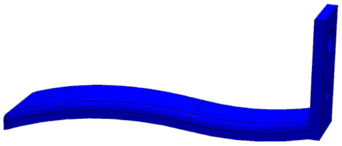

1x

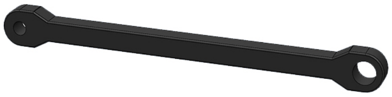

2x

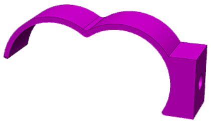

1x

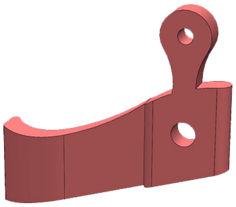

1x

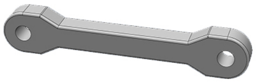

1x

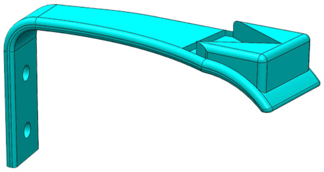

3x

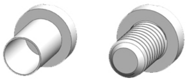

3/8" post-screws

1x

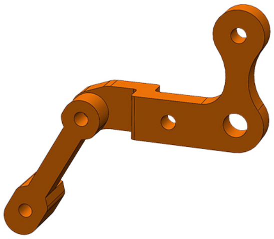

7x

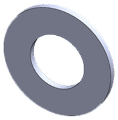

washers

1x

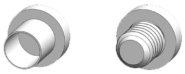

1/4" post-screws

1x

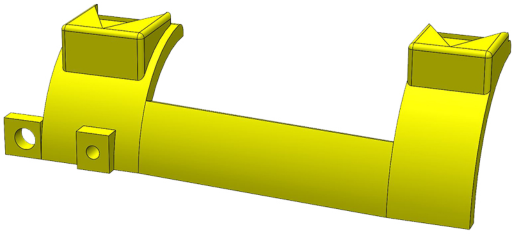

1

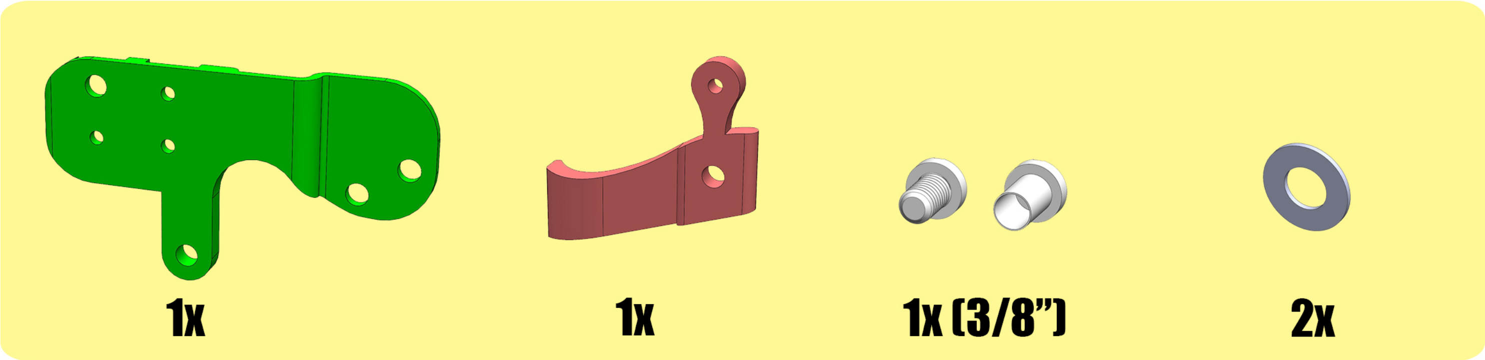

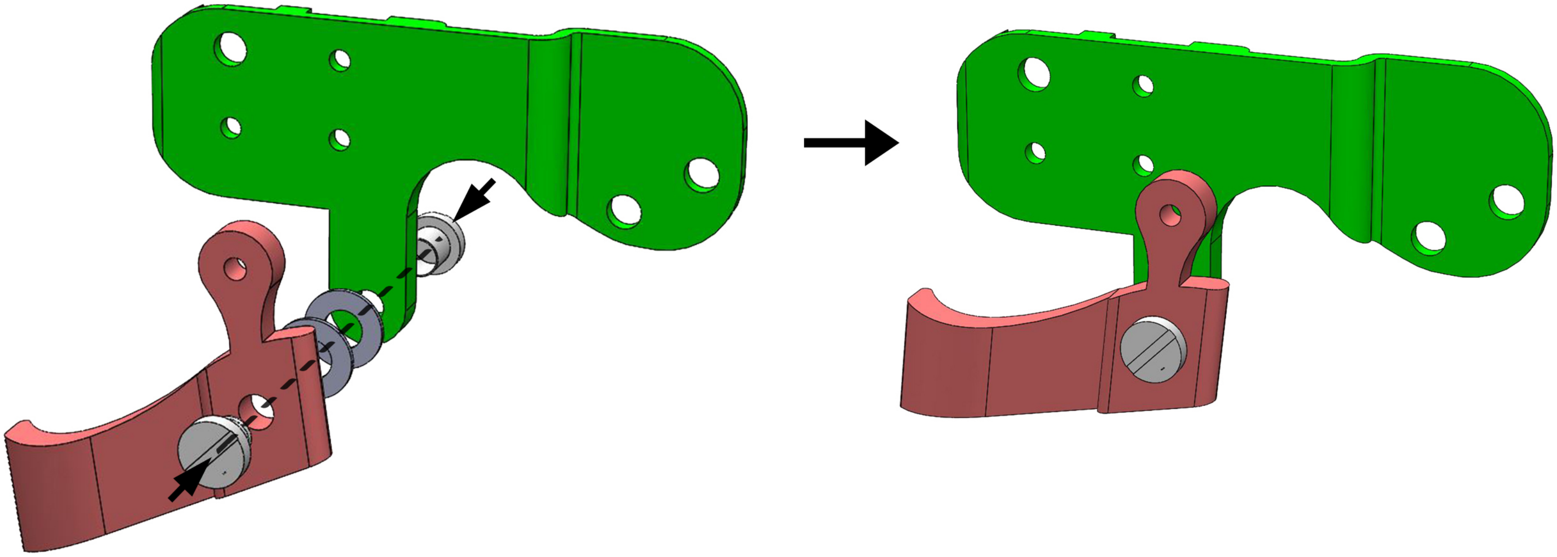

2

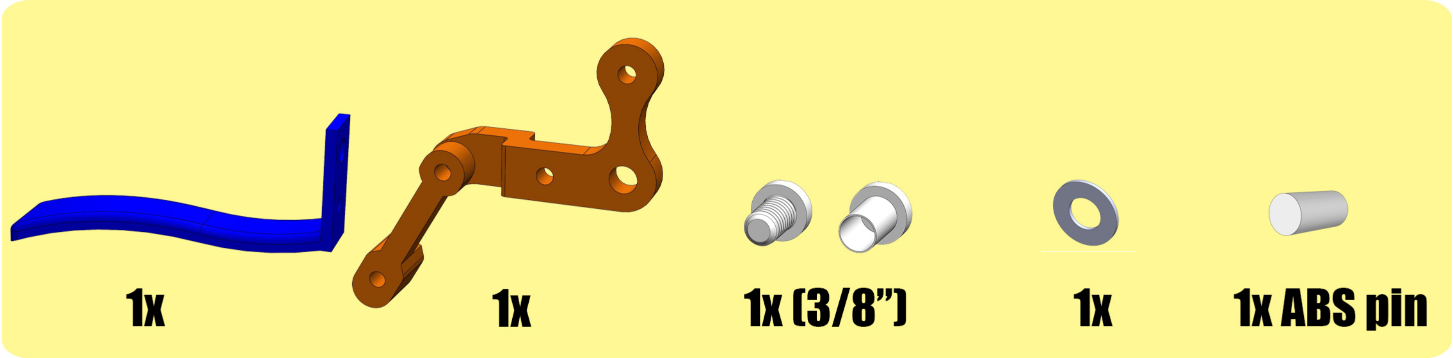

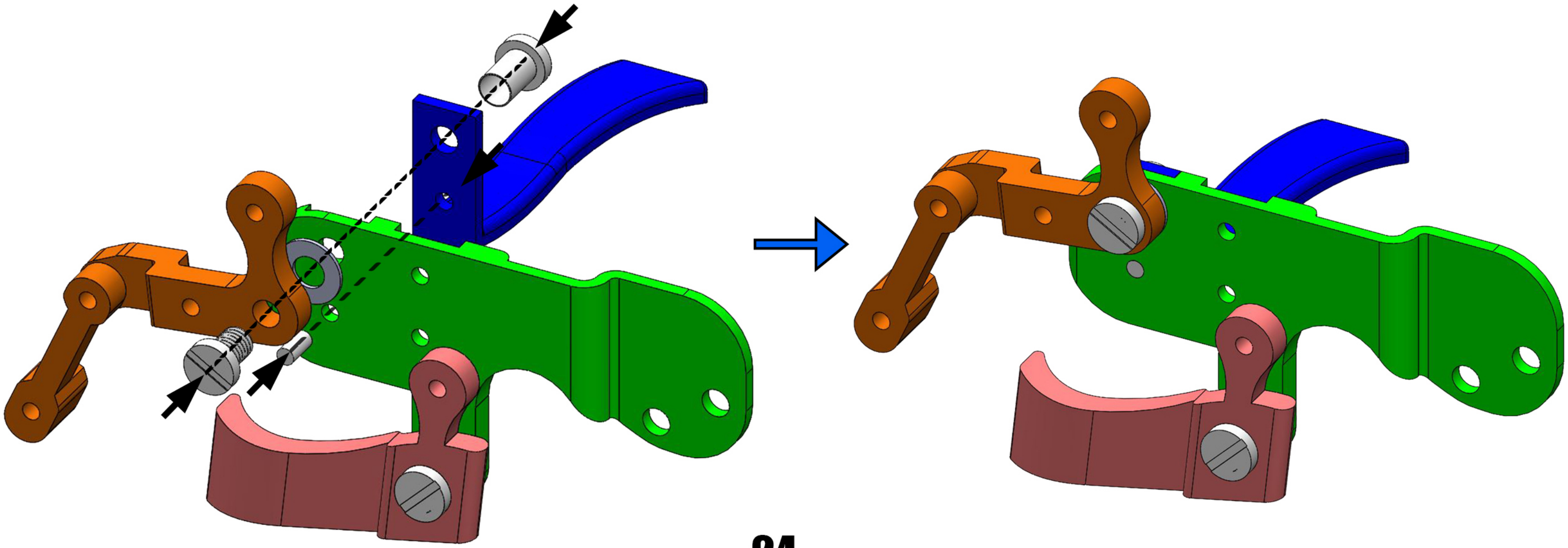

3

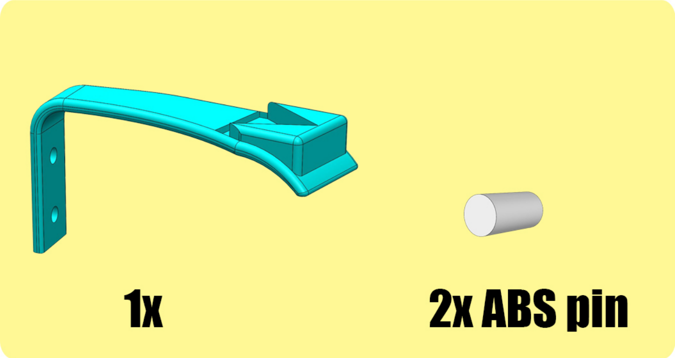

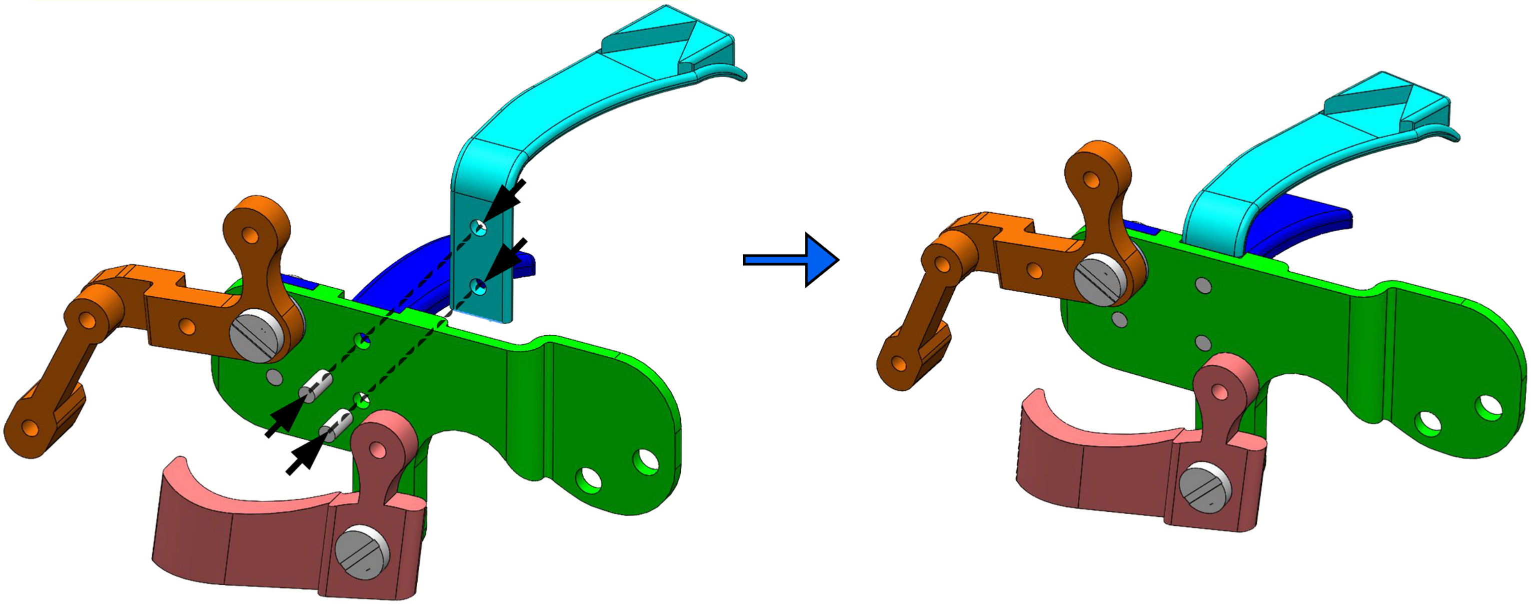

4

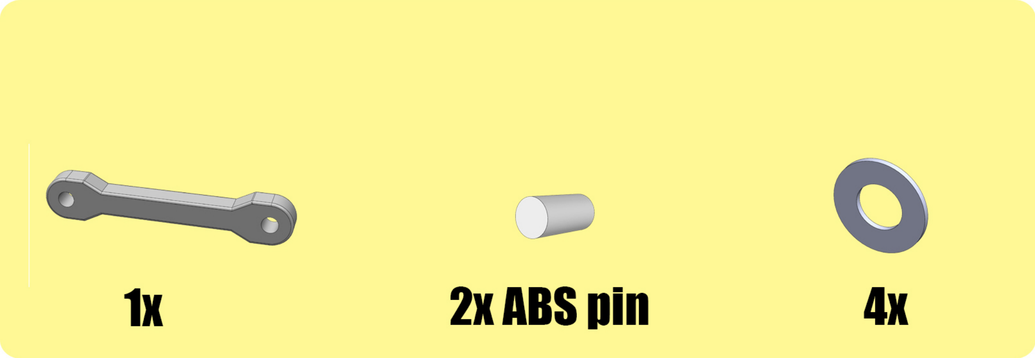

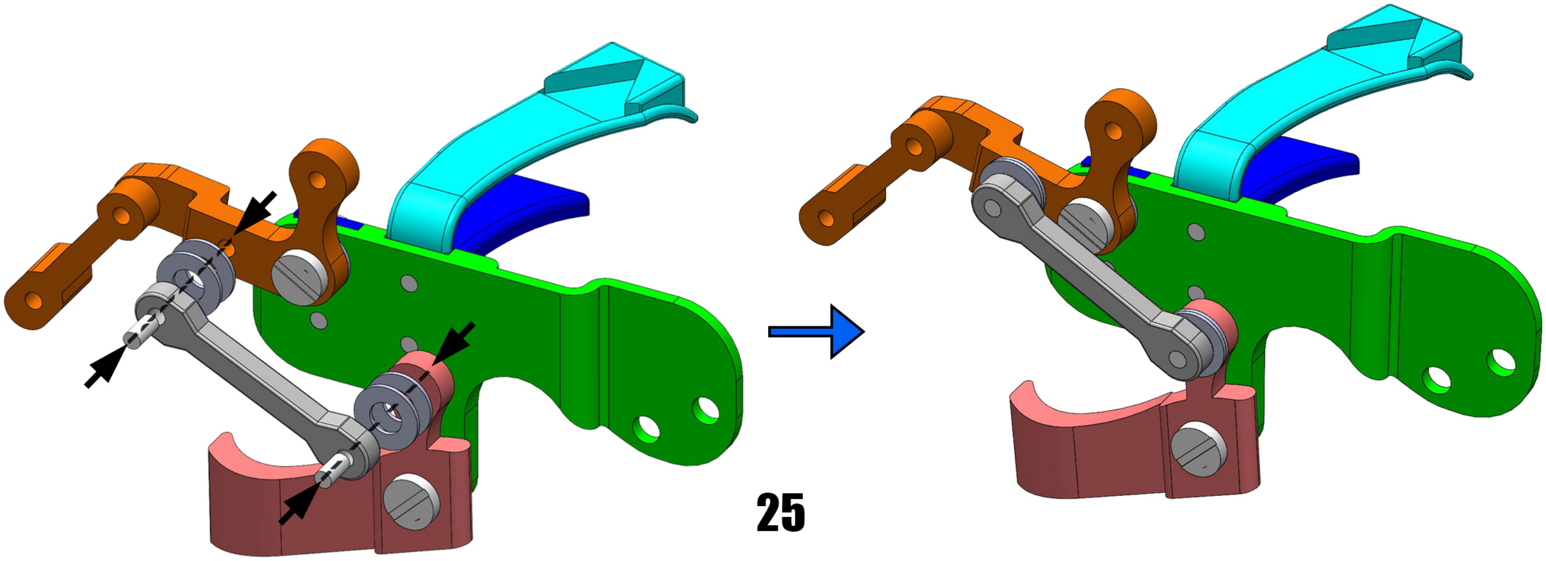

5

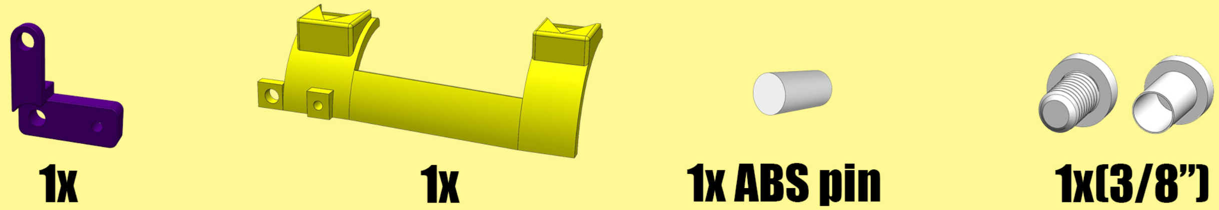

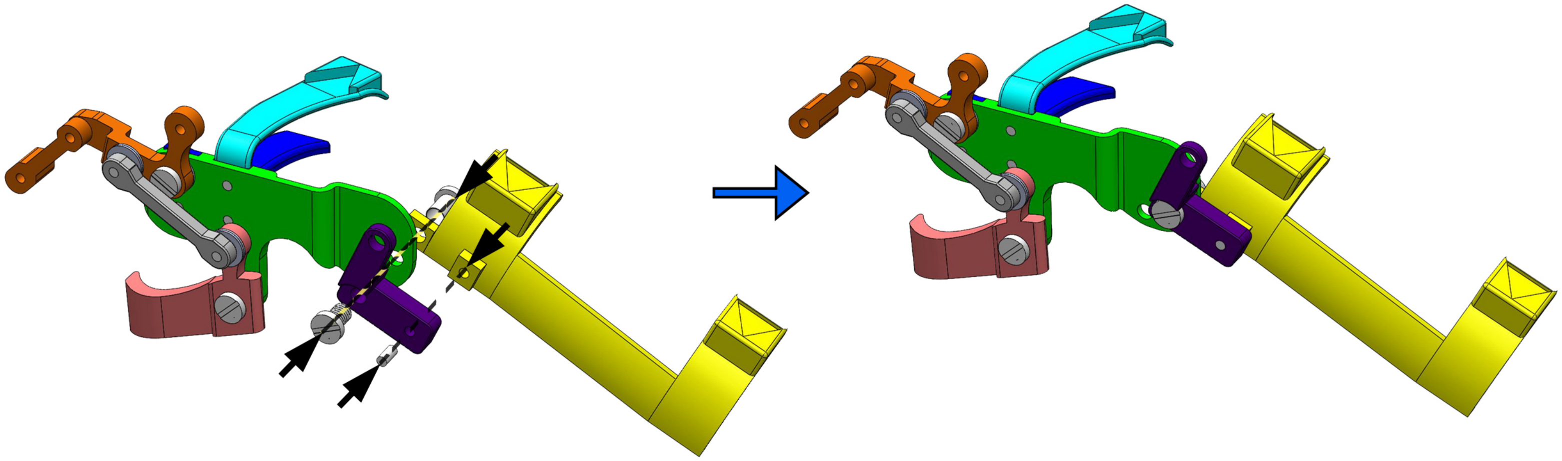

6

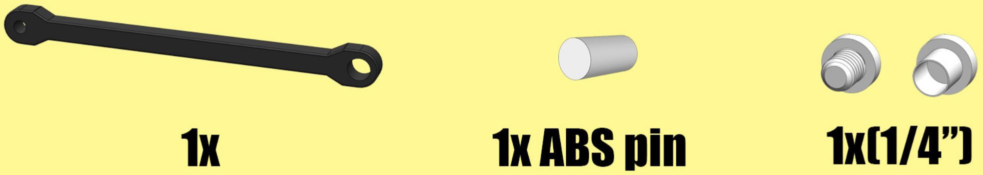

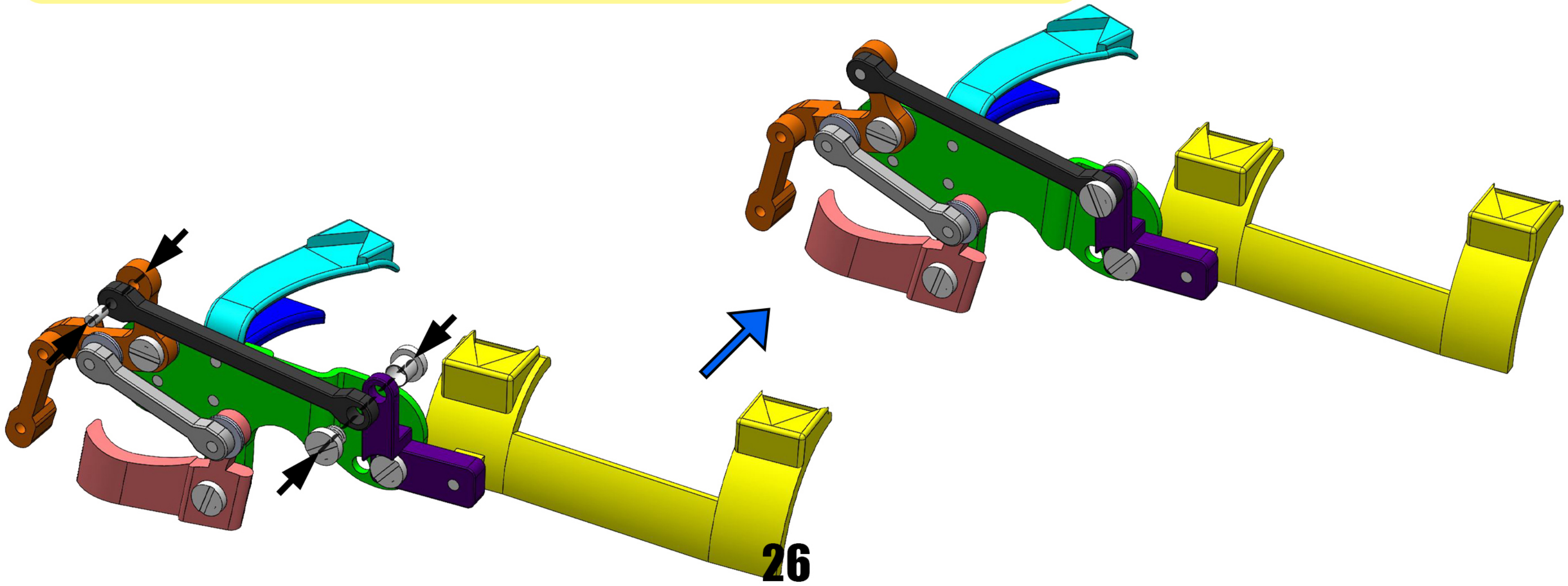

7

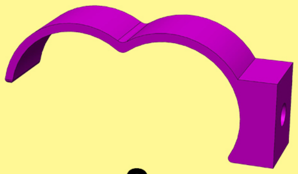

2x

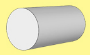

2x ABS pin

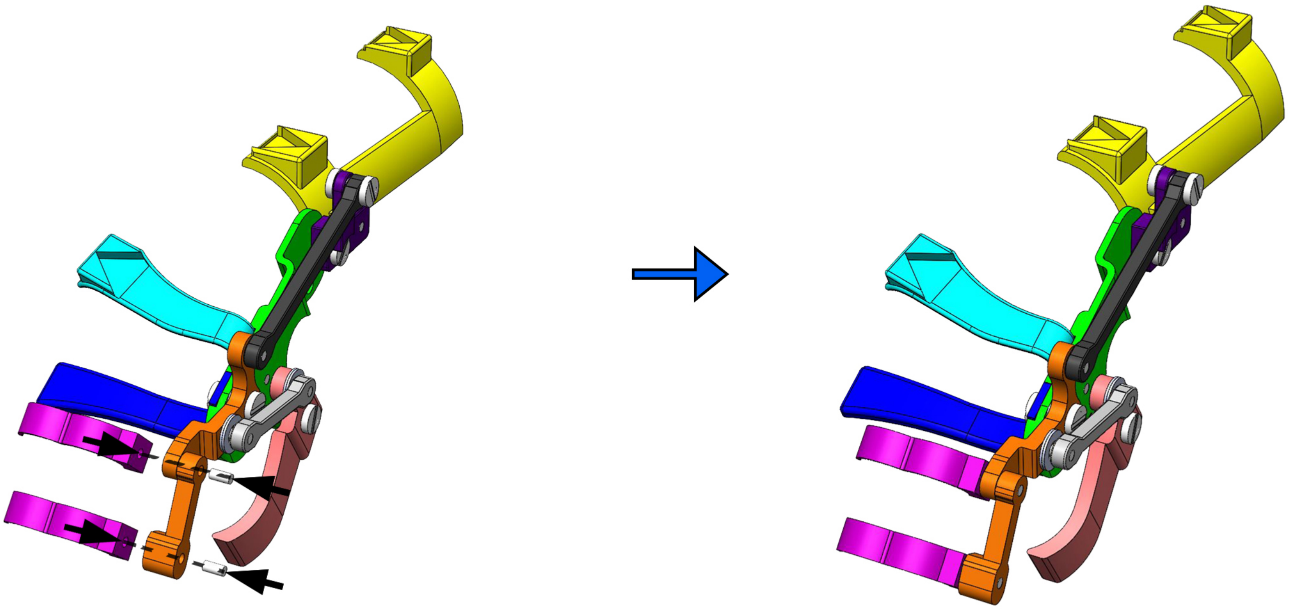

5.5 Post-Assembly

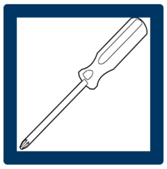

1. Adjust the tightness level of each post-screw. Make sure that the WDO still functions properly.

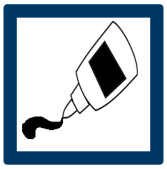

2. Apply thread-lock to the back of each post-screw and let it set for at least 20 mins. Make sure the thread-lock covers the threads.

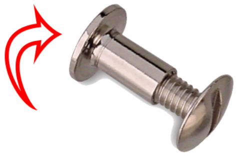

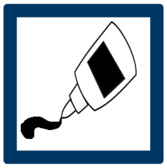

3. Glue the padding onto corresponding components. Use super-glue to ensure it is securely attached.

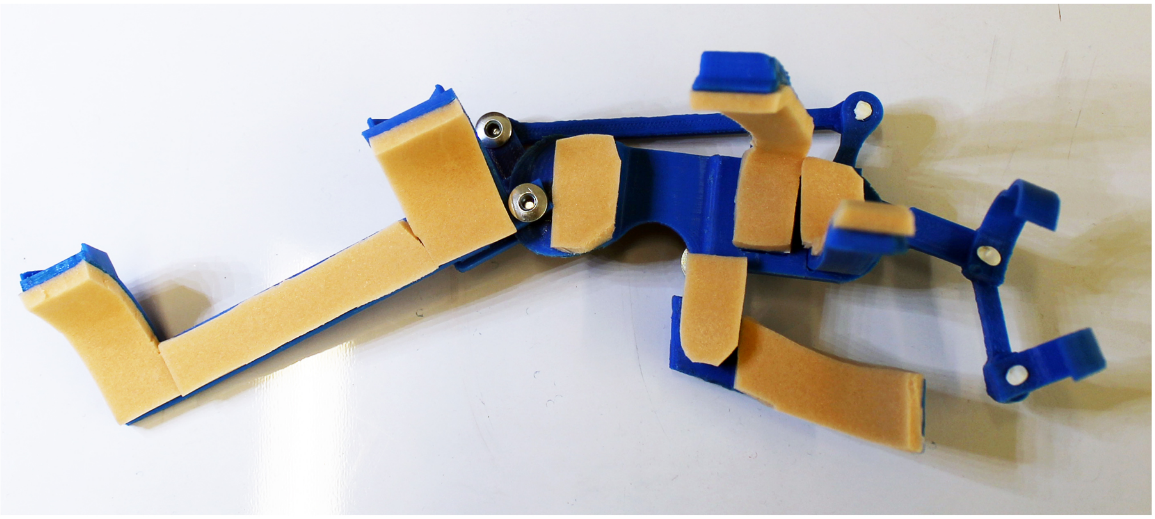

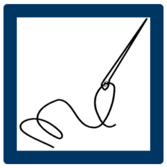

4. Using an industrial-strength thread, sew the 3D-printed rings onto the corresponding forearm and hand straps.

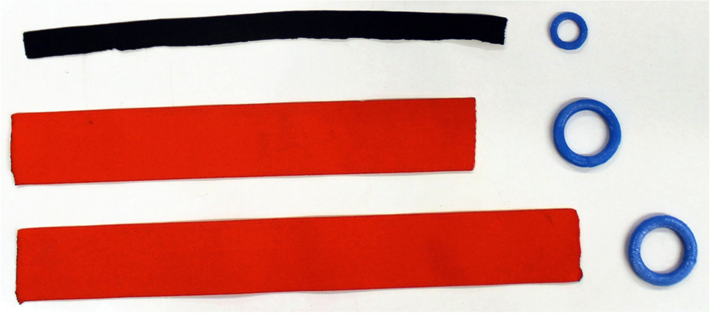

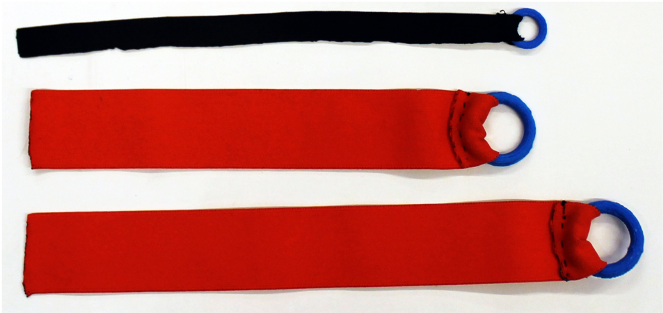

5. Prepare the self-adhesive magnets. The positive should go inside the triangular ridges on the dorsal and forearm pieces and the negative should go onto the triangle part.

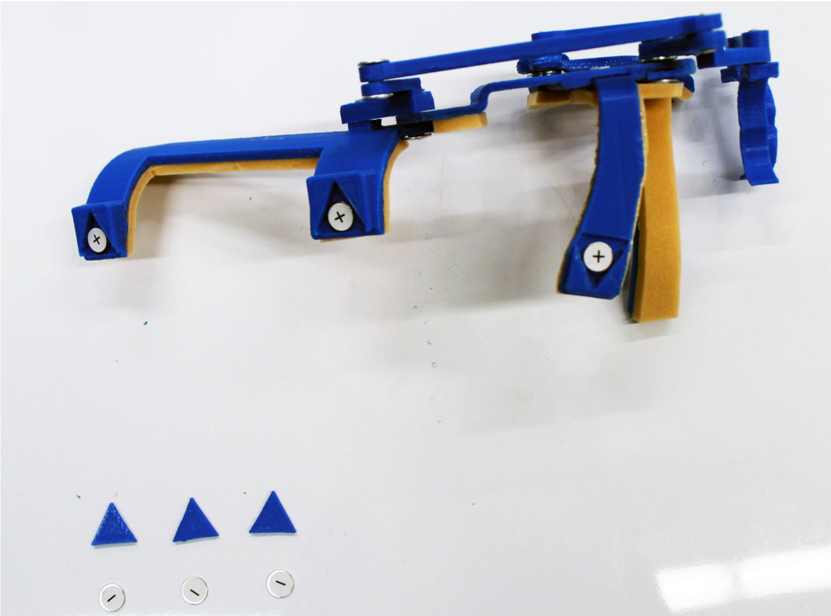

6. Superglue the magnets to their corresponding pieces. Be careful not to glue your fingers.

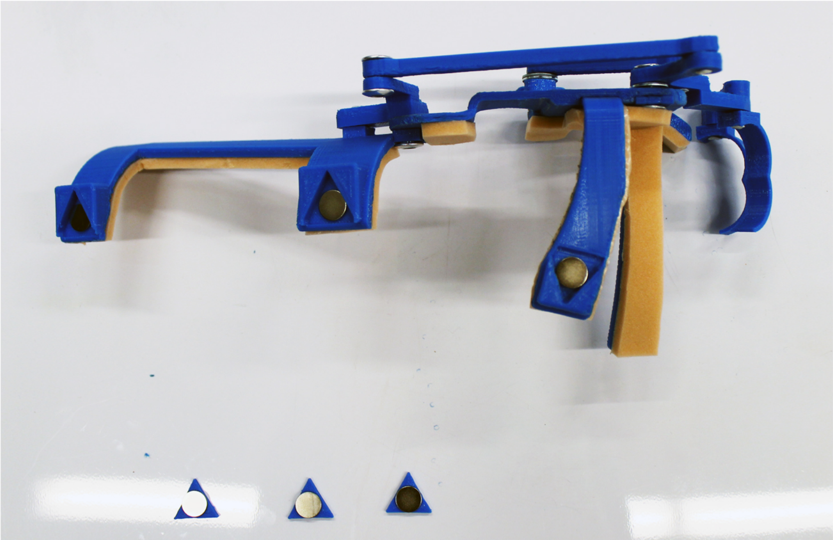

7. Using superglue, glue the forearm straps to the inside of the forearm piece, right onto the padding (see Step 8, page 30).

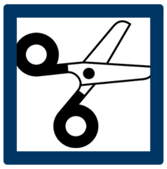

8. Cut a small hole on the hand strap approximately 10mm away from the edge without the ring. Attach the hand strap to the WDO with a 1/4” post-screw through the remaining hole in the *hand piece.*

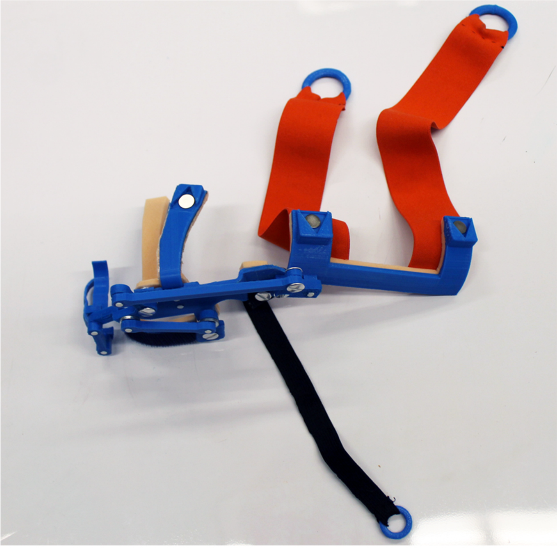

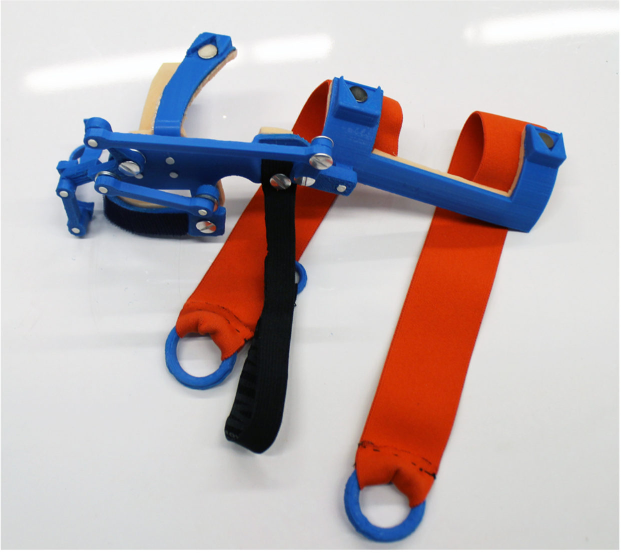

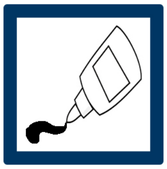

9. For this step, we recommend having the WDO user present. Glue the triangle pieces to the forearm and hand straps in the following way. The straps should not be too tight to prevent the blood flow or too loose to allow the WDO fall off the hand.

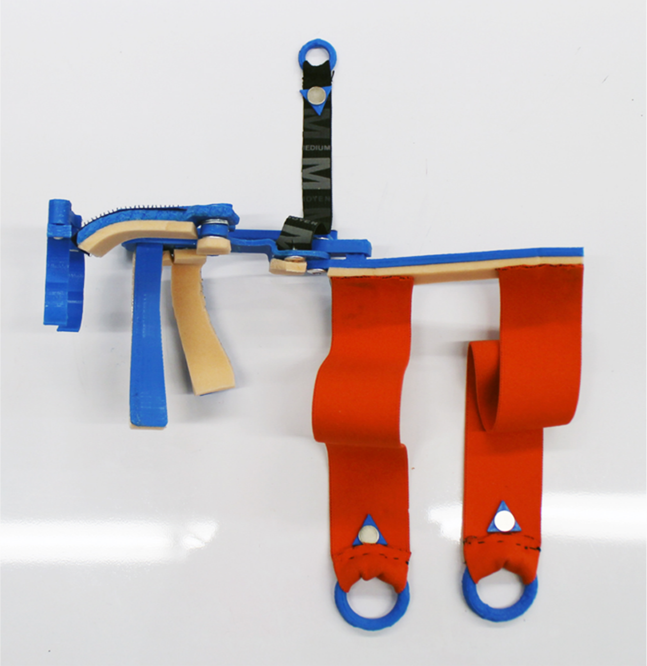

10. Wrap the thumb strap around the thumb piece as shown below.

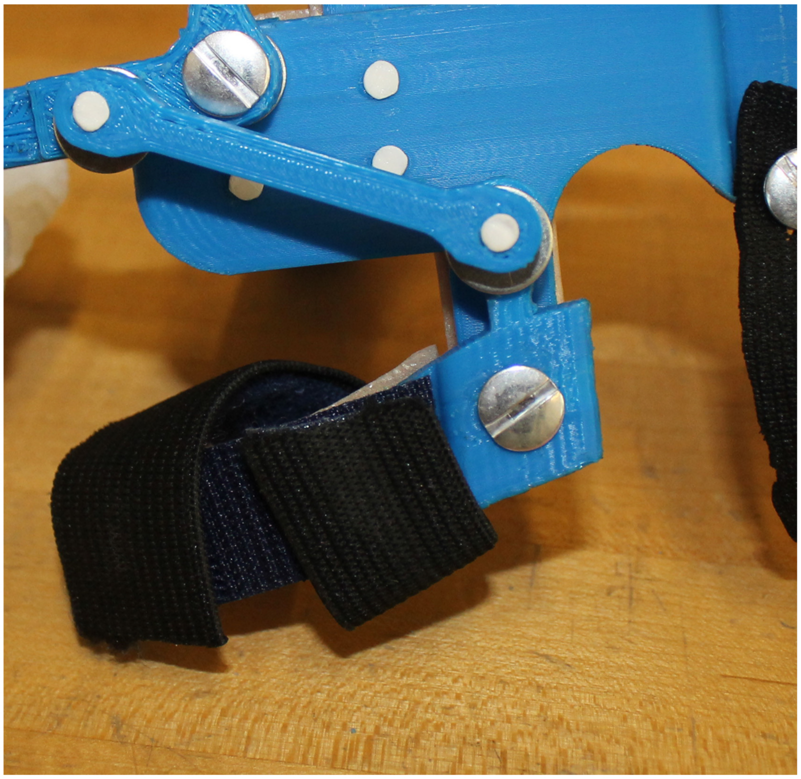

5.6 Finger Shell

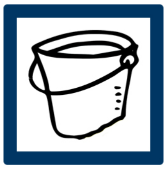

1. Prepare a pot of water at approximately 150F, scissors, Instamorph, heat gun, and hot glue gun.

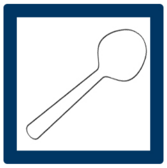

2. Place approximately a tablespoon of Instamorph into the pot of water and wait until white pellets turn clear and stick together (~2min).

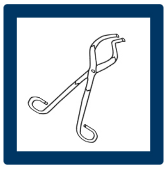

3. Using tongs or other tools, carefully remove from the pot and pat dry. First, flaten it out on a table and attempt to even it out to eliminate the presence of holes and uneven surfaces.

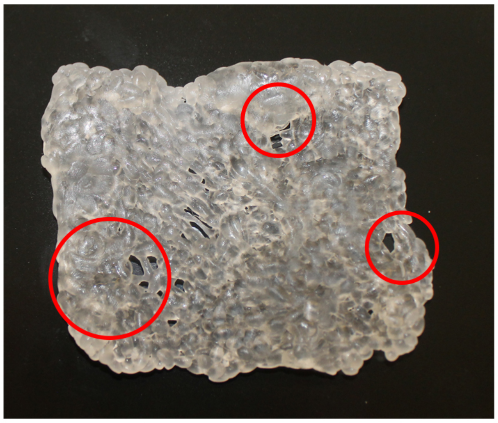

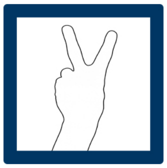

4. Wrap the clear plastic around the user's first and second digits. Asked them to keep their fingers in a three-jaw-chuck grasp as shown-below. Use scissors to cut any unnecessary material. Make sure finger pads are open and not covered.

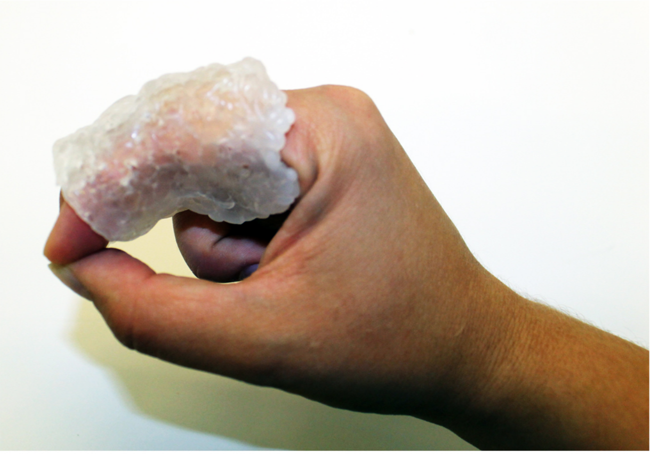

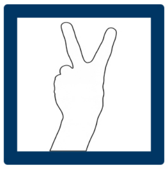

5. Hold the plastic in place until cool and solid. If you still see un-evenness on the surface, place the piece into the pot of hot water again and wait for it to turn clear before reshaping.

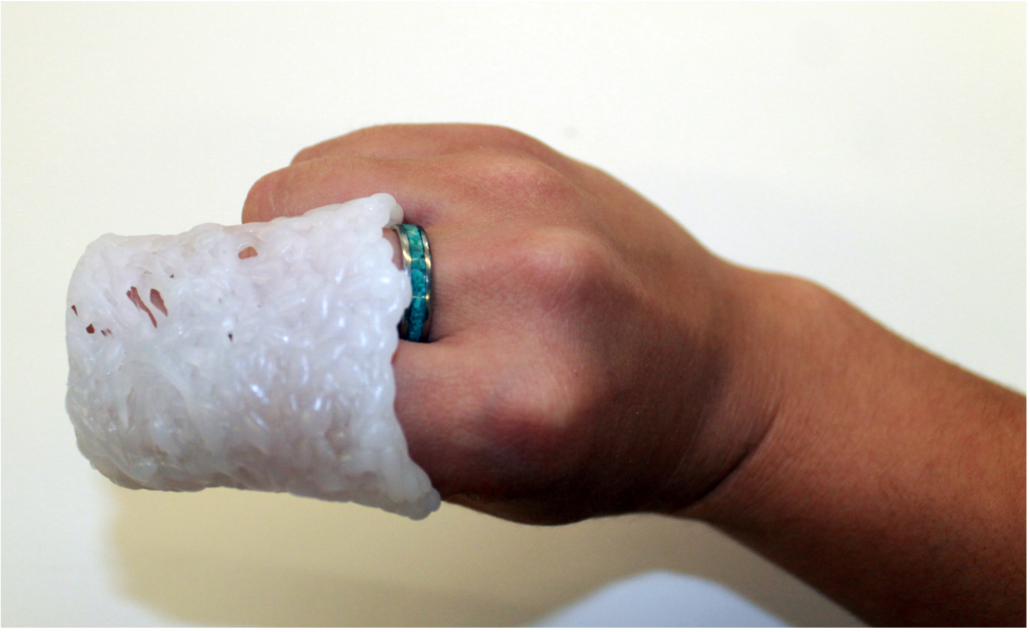

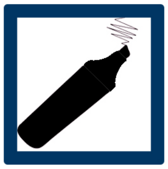

6. Now, place the finger shell under the finger rings on the WDO as shown below. It is best to perform this step with the user's hand inside the WDO. Mark the proper placement of the shell with

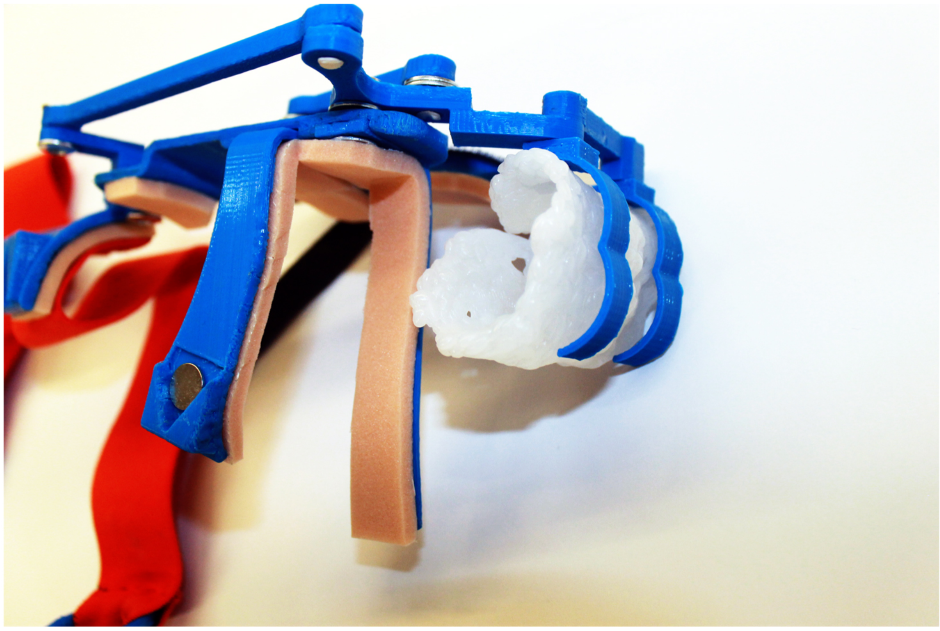

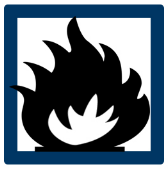

7. Without the user wearing the WDO, use a heat gun and slightly melt the finger rings. This will allow them to take the right shape of the shell. Be careful not to melt the shell itself. If you did, just turn off the heat gun and let it cool down.

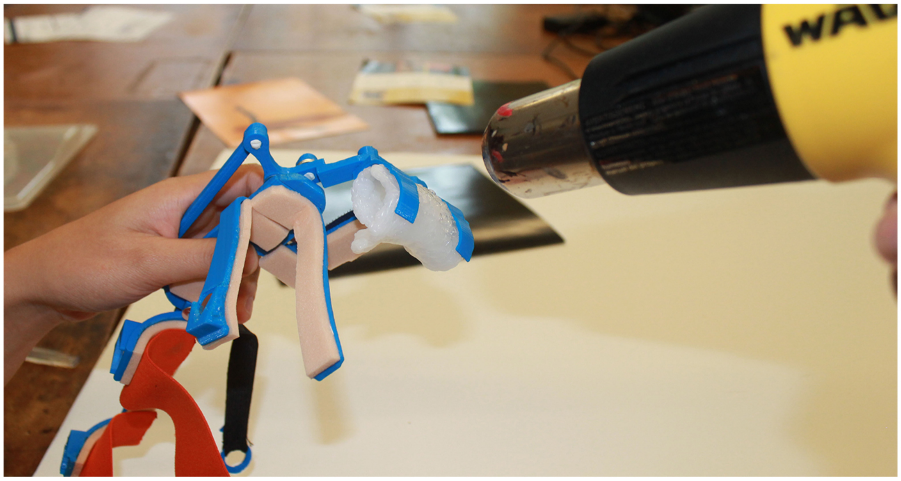

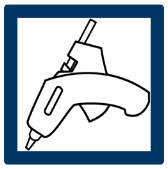

8. Using a hot glue gun, connect the shell to the inner surface of the rings.

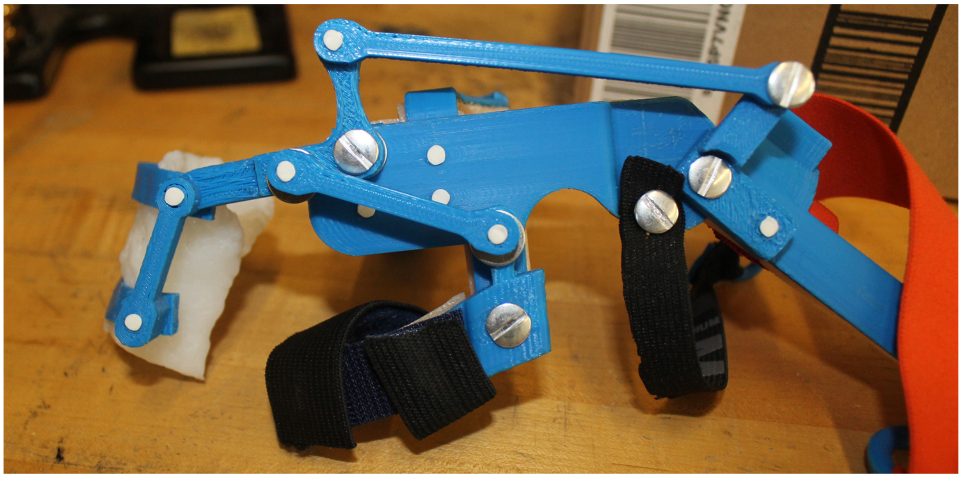

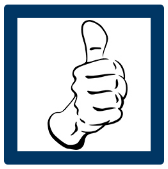

9. Ensure the WDO fits comfortably over the user's hand and does not cause any irritation or discomfort.
