# Supplementary figures and images for: Design of a 3D-printed, open-source wrist-driven orthosis for individuals with spinal cord injury
Source: PLoS One. 2018 Feb 22;13(2):e0193106. doi: 10.1371/journal.pone.0193106 (PMC5823450; doi:10.1371/journal.pone.0193106)

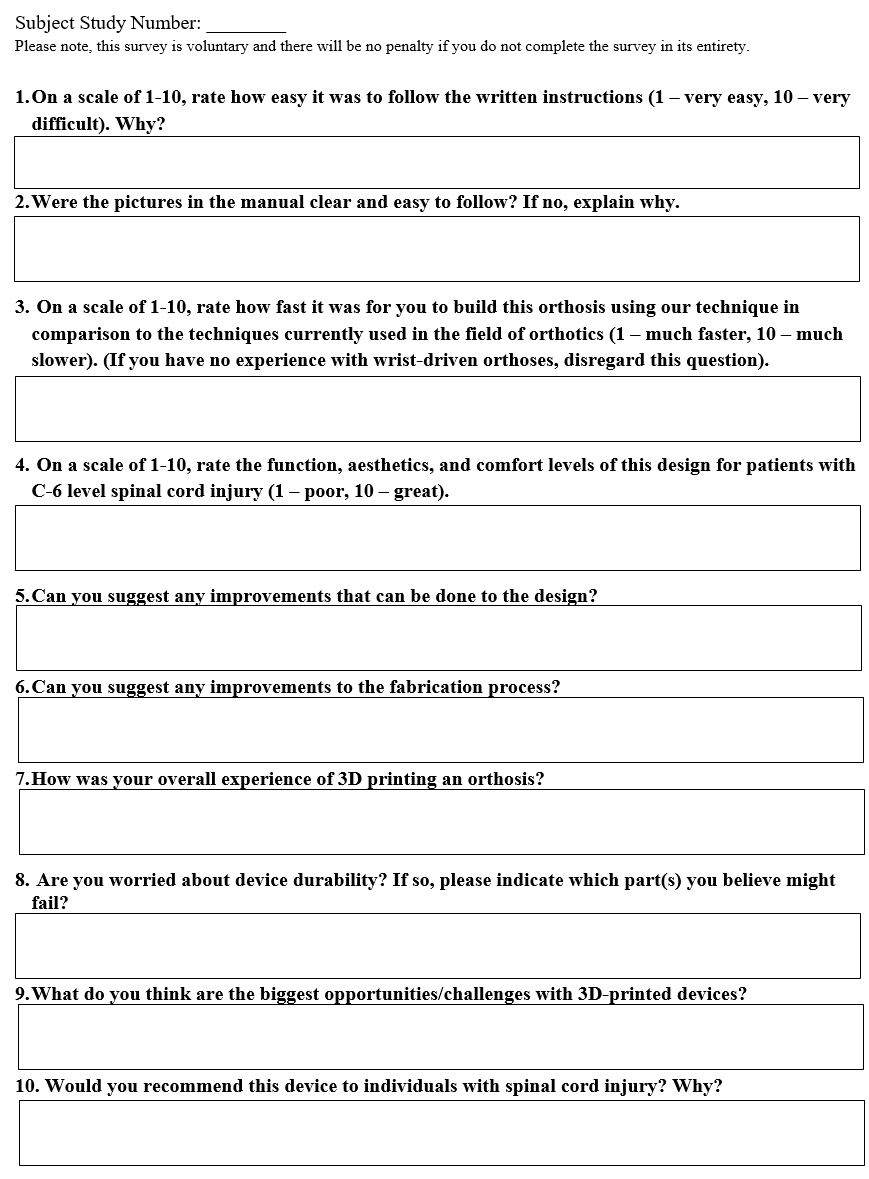

Supplement: S1 Survey — (JPG) [file pone.0193106.s002.JPG]

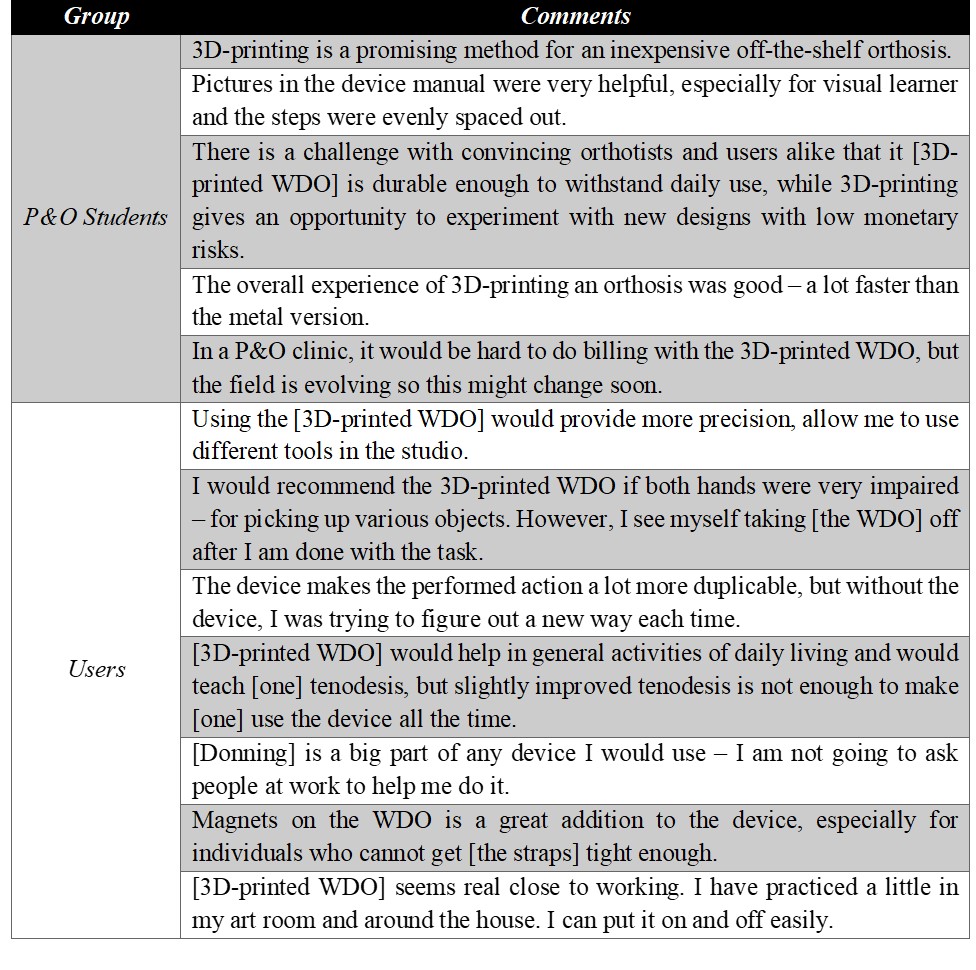

Supplement: S1 Table — (JPG) [file pone.0193106.s004.JPG]
